# Adsorption and Activation of CO_2_ on Nitride MXenes: Composition, Temperature, and Pressure effects

**DOI:** 10.1002/cphc.202100600

**Published:** 2021-10-13

**Authors:** Anabel Jurado, Kevin Ibarra, Ángel Morales‐García, Francesc Viñes, Francesc Illas

**Affiliations:** ^1^ Departament de Ciència de Materials i Química Física & Institut de Química Teòrica i Computacional (IQTCUB) Universitat de Barcelona c/Martí i Franquès 1-11 08028 Barcelona Spain

**Keywords:** carbon capture, nitride MXene, density functional calculations, charge transfer, kinetic phase diagram

## Abstract

The interaction of CO_2_ with nitride MXenes of different thickness is investigated using periodic density functional theory‐based calculations and kinetic simulations carried out in the framework of transition state theory, the ultimate goal being predicting their possible use in Carbon Capture and Storage (CCS). We consider the basal (0001) surface plane of nitride MXenes with M_*n*+1_N_*n*_ (*n*=1–3; M=Ti, Zr, Hf, V, Nb, Ta, Cr, Mo, and W) stoichiometry and also compare to equivalent results for extended (001) and (111) surfaces of the bulk rock‐salt transition metal nitride compounds. The present results show that the composition of MXenes has a marked influence on the CO_2_‐philicity of these substrates, whereas the thickness effect is, in general, small, but not negligible. The largest exothermic activation is predicted for Ti‐, Hf‐, and Zr‐derived MXenes, making them feasible substrates for CO_2_ trapping. From an applied point of view, Cr‐, Mo‐, and W‐derived MXenes are especially well suited for CCS as the interaction with CO_2_ is strong enough but molecular dissociation is not favored. Newly developed kinetic phase diagrams are introduced supporting that Cr‐, Mo‐, and W‐derived MXenes are appropriate CCS substrates as they are predicted to exhibit easy capture at mild conditions and easy release by heating below 500 K.

## Graphical abstract

**Kinetic phase diagrams** are introduced to predict the conditions at which nitride MXenes (M_2_N, M_3_N_2_, and M_4_N_3_) capture CO_2_. The factors affecting CO_2_ interactions are analyzed together with the implications for carbon capture and storage.

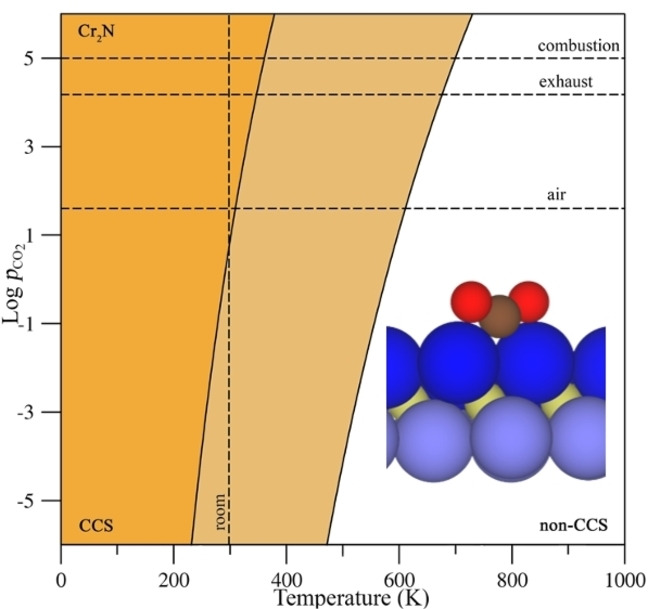

## 1 Introduction

The increase of the carbon dioxide (CO_2_) concentration in the Earth's atmosphere, mainly arising from burning fossil fuels for various applications,^[1]^ is undoubtedly the responsible for the continuous global warming through the well‐understood greenhouse effect.^[2]^ This implies ocean acidification, polar ice caps and mountain glaciers melting, higher sea levels, and extreme weather events. These will all contribute to hamper the Earth's ability to support life and represents a serious challenge to the existing biodiversity.^[3]^ Not surprisingly, the political agenda of many countries involve a roadmap to a low‐carbon scenario that involves several strategies such as reducing emissions and eliminating emissions, among others as recently reviewed by Hepburn *et al*.^[4]^ Regarding CO_2_ sequestration, Carbon Capture and Storage (CCS) and the subsequent utilization (Carbon Capture and Usage – CCU) technologies are currently the focus of considerable research endeavors both from experiments^[5]^ or tackled by computational modeling.^[6]^ CCS is regarded as a necessary short‐term bridge before CCU technologies mature, which needs to be improved for its efficiently implementation in a global scale. Apart from direct CO_2_ sequestration through biomass or geological sinks,^[4]^ considerable endeavors have been devoted at exploring novel materials for CO_2_ abatement as well as to optimize the involved chemistry.^[7]^ Amine‐based solutions, ionic liquids, or solid adsorbents seem to be among the mature approaches, although with some environmental concerns due to their toxicity and other harmful issues. Other investigated materials include pure metals, metal oxides, graphene derived materials, zeolites, and Metal Organic Frameworks (MOFs).[^8^, ^9^] In short, different strategies have been proposed for efficient CO_2_ conversion.[^6^, ^10^] However, the performance of many of these materials is far from optimum because of a low ability to adsorb, and eventually activate, CO_2_, thus involving harsh conditions.

In the search for alternative substrates for CO_2_ sequestration and activation, MXenes can step in because they have shown a sufficient strong way to adsorb CO_2_.[^11^, ^12^, ^13^, ^14^, ^15^, ^16^] This acronym, reminiscent of graphene, defines a new family of 2D materials that has rapidly grown since the isolation of Ti_3_C_2_T_*x*_, the first member of the family, in 2011, *vide infra*.^[17]^ This discovery generated a great expectation in several fields of applicability such as energy, catalysis, biomedics, electronics, and environmental applications, to name just a few.^[18]^ All MXenes exhibit a M_*n*+1_X_*n*_T_*x*_ general formula, where M is as early transition metal, X=C or N, *n* defines the MXene width (normally *n*=1–3), and T_*x*_ represents one of the common terminations, most often −O, −OH, −F, or −H,^[19]^ that are inherent to their synthesis process. The synthesis of MXenes follows a top‐down approach by a selective disassembly of the MAX phase precursors; see the recent review by Zhang *et al*.^[20]^ In the MAX phase, A stands for Al, Si, or other elements in *p*‐block. The removal of A from the MAX phase is achieved by using different chemical etchants. Initially, the hazardous hydrofluoric acid (HF) was selected as etchant agent,^[17]^ but F‐free and Lewis acid‐based routes have been very recently reported.[^21^, ^22^] The broad number of applications of these materials is associated to the composition that depends on selecting M and X elements, and the number of atomic layers, *n*, controlled by selecting the appropriate MAX precursors and, finally, the surface terminating species; T_*x*_, whose presence depends on the employed etchant agent and on the followed synthesis protocol. These three structural variables offer great opportunities for tailoring the surface chemistry and properties of MXenes.^[23]^ We must emphasize that, although the usual synthesis lead to functionalized MXenes, recent work reported experimental protocols that that provide bare MXenes without T_*x*_,[^15^, ^21^] which opens the way to study the intrinsic chemistry of MXenes as well as their possible applications.

The bi‐dimensional (2D) structural nature along with its carbide and/or nitride chemical composition suggest that MXenes may occupy a privileged position within the field of heterogeneous catalysis and electrocatalysis.^[24]^ This view is supported by the computational prediction that pristine MXene carbides with M_*n*+1_C_*n*_ formula have a strong capability for activating and converting CO_2_,^[11]^ a forecast later experimentally confirmed,^[15]^ reinforcing the potential of MXenes for CO_2_ abatement, with promising CO_2_ uptakes of *ca*. 8–12 mol kg^−1^ on individual MXene sheets. Further studies investigating CO_2_ adsorption and desorption isotherms on MXenes such as Ti_3_C_2_T_*x*_ and V_2_CT_*x*_, carried out at 298 K under 0–4 MPa, confirmed that the slit‐like shape interlayer space generated during their synthesis is responsible for the CO_2_ storage.^[16]^ The promising results observed on MXene carbides calls for research on their counterpart nitrides.

This family has proven difficult to synthesize because, compared to the carbide family, they exhibit lower cohesive and higher formation energies,[^25^, ^26^] added to the fact that aqueous HF etchant solution are not suitable for isolating M_*n*+1_N_*n*_T_*x*_.^[27]^ Fortunately, novel synthetic routes as those mentioned earlier,[^21^, ^22^] have contributed to overcome this drawback making the synthesis of nitride MXenes feasible.[^28^, ^29^] At this point, investigating the performance nitride MXenes for CO_2_ activation becomes timely. Indeed, a previous computational study employing Density Functional Theory (DFT) methods indicated that bare M_2_N (M=Ti, Zr, Hf, V, Nb, Ta, Cr, Mo, and W) systems are suitable for CO_2_ capture and activation with predicted adsorption energies even higher than those predicted for the counterpart carbides, using the same computational approach.^[13]^ These promising results required further studies aimed at investigating several open issues such as the effect of the MXene thickness and, more importantly, to identify the temperature and CO_2_ partial pressure conditions at which such materials are appropriate for CCS. Both issues are addressed in the present work, the reported results providing a guide nitride MXenes on which the activation of CO_2_ occurs at mild conditions.

### 1.1 Computational Models and Methods

Extended surface models are employed to represent the basal (0001) plane of bare nitride MXenes with formula M_*n*+1_N_*n*_, *n* running from 1 to 3, and M being an early transition metal, including Ti, Zr, Hf, V, Nb, Ta, Cr, Mo, and W. The M_2_N surface model is built by removing the A element of the MAX phase followed by a full structural optimization. The resulting M−N−M sandwich‐like configuration provides a realistic model for bare MXenes with stoichiometry M_2_N and constitutes the starting point for building up thicker MXenes with general M_3_N_2_ and M_4_N_3_ formula by simply altering M and N layers following the ABC stacking sequence. In all cases, the generated M_3_N_2_ and M_4_N_3_ structures were fully relaxed prior to study the CO_2_ adsorption. A *p*(3×3) supercell is always used to minimize the lateral interaction between adsorbed CO_2_ molecules in periodically replicated images. Because, as indicated below, the calculations involve an intrinsically periodic plane wave basis set, it is necessary to include a vacuum width to avoid a spurious interaction between the periodic replicas in the direction perpendicular to the surface. Thus, a width of 10 Å is selected as it is sufficient to obtain numerically converged results, as discussed in detail in previous works.[^11^, ^13^] For comparison, the (001) and (111) surfaces of the corresponding bulk face‐centered cubic (*fcc*) Transition Metal Nitrides (TMNs) have been also considered. The corresponding slab models are built following the standard approach. From a structural viewpoint, the planes perpendicular to the [001] direction maintain the 1 : 1 stoichiometry of the bulk TMN, whereas those perpendicular to the [111] direction feature alternating M or N atomic layers alternate generating either M or N surface terminations. To properly compare to the MXene (0001) surfaces, only the M‐terminated layer perpendicular to the [111] direction is considered. By moving from 2D to extended surfaces of bulk TMNs we will be able to firmly establish the influence of thickness on the adsorption and subsequent activation of CO_2_.

To investigate the properties of the surfaces and their interaction with CO_2_, we rely on first‐principles periodic DFT based calculations. From the obtained results we analyze the potential of these MXenes in CCS strategies. The total energy of the explored M_*n*+1_N_*n*_ (0001) surfaces −M=Ti, Zr, Hf, V, Nb, Ta, Cr, Mo, and W− with *n*=1–3, as well TMN (001) and (111) ones without or with CO_2_ has been optimized using the Perdew‐Burke‐Ernzerhof (PBE)^[30]^ exchange‐correlation functional with the dispersion forces effects included through Grimme's D3 method,^[31]^ as implemented in the Vienna *Ab Initio* Simulation Package (VASP) code.^[32]^ A *p*(3×3) supercell is used for the TMN (111) surfaces, and a *c*(2√2×2√2)P45° for the TMN(001) ones, as previously used in the past,[^11^, ^33^] and having a similar number of surface metal centers; nine or eight, respectively, and so, with a comparable molecular coverage. An expansion of the valence electron density in a Plane‐Wave (PW) basis set with a kinetic energy cut‐off of 415 eV is used to solve the Kohn‐Sham (KS) equations and the interaction between the valence electron density and the core electrons is taken into account by means of the Projector Augmented Wave (PAW) method;^[34]^ the numerical integrations in the reciprocal space were carried out using a Monkhorst‐Pack^[35]^ grid of 5×5×1 special **k**‐points is. A denser 9×9×1 grid of **k**‐points is used to study the TMN surfaces. The geometry optimizations reach the convergence when the forces acting over the nuclei are all below 0.01 eV Å^−1^. This computational setup ensures converged results up to 1 meV in the calculated adsorption energy.

The CO_2_ adsorption energy on the different MXene (0001), and MN (001) and (111) substrates is computed as $E_{ads}=\;E_{CO_{2}@substrate}-\left(E_{CO_{2}}+\;E_{substrate}\right)+\;\Delta E_{ZPE}$ where $E_{CO_{2}@substrate}$ corresponds to the total energy of the CO_2_ anchored over either MXene and TMN surfaces, $E_{substrate}$ and $E_{CO_{2}}$ stand for the total energy of relaxed pristine substrates and of an isolated CO_2_ molecule, respectively, and $\Delta E_{ZPE}$ includes the difference in the zero‐point energy of each energetic contribution obtained assuming the harmonic approximation and decoupling of surface phonons and adsorbate vibrations. The vibrational frequencies were obtained by diagonalization of the corresponding block of the Hessian matrix with elements computed as finite difference of analytical gradients with displacements of 0.03 Å. The definition of $E_{ads}$ above implies that negative values correspond to exothermic adsorptions. Spin polarization was not considered as this does not significantly affect $E_{ads}$ of the M_*n*+1_N_*n*_ (0001) surfaces as recently reported by some of us.^[36]^

The results from the DFT calculations are next used to compute adsorption, *r*_ads_, and desorption, *r*_des_, rates at different pressure and temperature conditions. Following a previous work on CO_2_ adsorption/desorption on transition metal carbides,^[33]^ it is assumed that CO_2_ adsorption is a non‐activated process and can be calculated from the well‐known Hertz‐Knudsen equation as in Eq. 1,

(1) $$r_{ads}=\frac{S_{0}\cdot p_{{CO}_{2}}\cdot A}{\sqrt{2\pi \cdot m\cdot k_{B}\cdot T}},$$

where *S*_0_ is the initial sticking coefficient, $p_{{CO}_{2}}$ corresponds to the CO_2_ partial pressure above the surface, *A* stands for the area of an active adsorption site and *m* is the mass of the adsorbed molecule. A conservative value of *S*_0_=0.40 is selected for our study following a previous analysis where the CCS were investigated on transition metal carbides and MXenes.[^11^, ^13^, ^33^] On the other hand, the rate of desorption, *r*_des_, is estimated from Transition State Theory (TST)^[37]^ assuming that the transition state for desorption is close enough to the final state so that the energy barrier can be estimated from the desorption energy. Therefore, the (negative) *E*_ads_ is used to estimate *r*_des_ as in Eq. 2

(2) $$r_{des}=\nu_{des}\cdot exp\left(\frac{E_{ads}}{k_{B}\cdot T}\right);\;\nu_{des}=\frac{k_{B}\cdot T\cdot q_{trans,2D}^{gas}\cdot \;q_{rot}^{gas}\cdot \;q_{vib}^{gas}}{q_{vib}^{ads}}\;,$$

with $\nu_{des}$ corresponding to the partition function of the molecule in a late 2D transition state. Here, the calculated harmonic frequencies are used to estimate the partition function of the adsorbed species whereas all degrees of freedom are considered for the gas phase CO_2_ molecule. Further details can be found in Refs. [13,33].

Following this strategy, multiscale simulations are carried with the aim to shed light on the conditions at which nitride MXenes may be appropriate for CCS. These are established by analyzing the crossover between CCS and non‐CCS crossover that is defined by a temperature and a CO_2_ partial pressure ($p_{CO_{2}})$ at which *r*_ads_=*r*_des_. This dynamic equilibrium situation allows us to derive what we refer to as kinetic phase diagrams, a newly developed tool introduced previously by some of us,^[12]^ where the kinetic term indicates that the phase diagrams are directly built from rates rather that from thermodynamic equilibrium arguments.

## 2 Results and Discussion

### 2.1 Adsorption Trends

**Figure 1:**
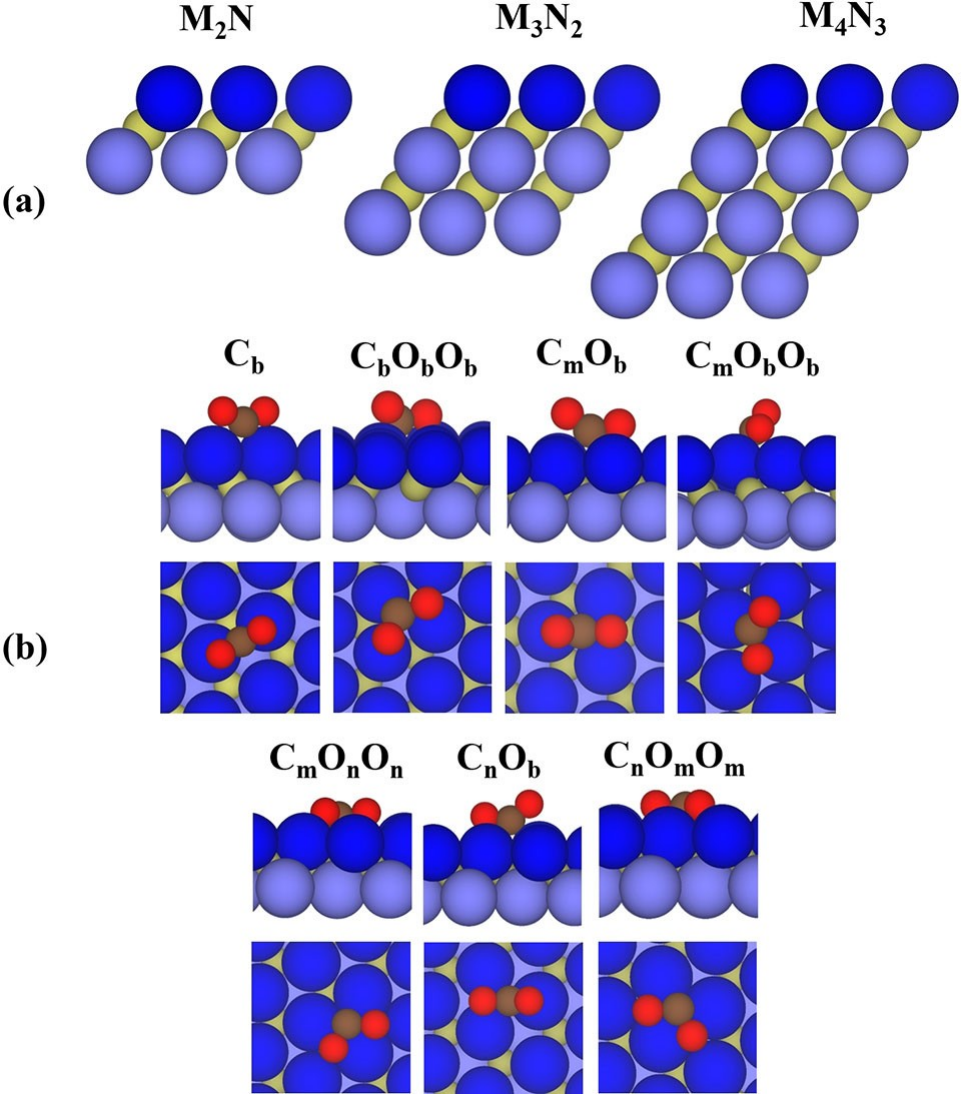
(a) Atomic structure of nitride MXene (0001) surfaces with formula M_*n*+1_N_*n*_ (*n*=1–3) where dark and light blue spheres represent the M upper, intermediate, and bottom layers, respectively, and the inner nitrogen layer is depicted by dark yellow spheres. The M upper layer contains the sites CO_2_ adsorption takes place; (b) side and top views of CO_2_ adsorbed on C_b_, C_b_O_b_O_b_, C_m_O_b_, C_m_O_b_O_b_, C_m_O_n_O_n_, C_n_O_b_, and C_n_O_m_O_m_ sites of MXene (0001) surfaces. In this notation, the capital letters (C or O) indicate the atoms of the CO_2_ molecule that interact directly with the MXene surfaces, and the subindices indicate the bridge (b), metal (m) or nitrogen (n) hollow surface sites closest to C or O atoms.

We start this section by analyzing the variation of the CO_2_ adsorption energy with respect to the composition and thickness of nitride MXenes, see Figure 1a. In this analysis, we explored the Potential Energy Surfaces (PESs) corresponding to different possible adsorption sites and different orientations of the CO_2_ molecule. The most favorable conformations for CO_2_ adsorption on the investigated nitride MXenes are displayed in Figure 1. We point out that these configurations are favorable at least in one of investigated MXenes regardless of the thickness and the composition. Similar configurations were also obtained for CO_2_ adsorption on the M‐terminated TMN (111) surfaces whereas different conformations were found on the TMN (001) surfaces as depicted in Figure S1 in the Supporting Information (SI).

**Figure 2:**
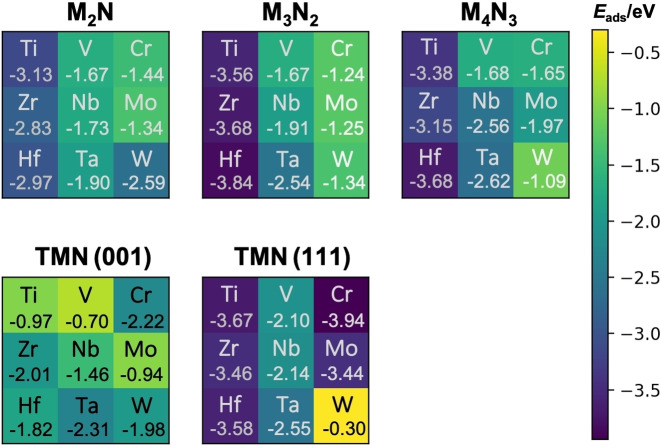
CO_2_ adsorption energies, *E*_ads_, on MXene and TMN surfaces. The scheme colors indicate the strength of the interaction between CO_2_ and the substrates, given in eV. Dark blue and yellow correspond to the largest and weakest interaction. Further details can be found in the SI. Values below the label of atoms correspond to the most favorable adsorption energies on the corresponding MXene and TMN surfaces.

Previous DFT calculations using PBE‐D3 approach have recently reported highly exothermic CO_2_ adsorption energies on bare M_2_N MXenes with values ranging from −1.03 (Mo_2_N) to −3.13 eV (Ti_2_N).^[13]^ This study also predicted that nitride *d* ^2^‐MXenes (Ti, Zr, and Hf) have the strongest adsorption (*ca*. −3.0 eV) followed by *d* ^3^‐V, Nb, and Ta (*ca*. −1.8 eV), and *d* ^4^‐Cr, Mo, and W (*ca*. −1.4 eV). Here, the case of Cr_2_N is an outlier being its behavior like a *d* ^3^‐metal. These previous results together to those obtained here for the thicker M_3_N_2_ and M_4_N_3_ MXenes indicate that nitride MXenes with M_3_N_2_ stoichiometry have similar or even larger CO_2_‐philicity than the M_2_N counterparts, see Figure 2. In particular, nitride *d* ^2^‐MXenes with M_3_N_2_ stoichiometry feature CO_2_ adsorption energies above −3.5 eV. Interestingly, no significant changes are found when moving to M_4_N_3_ nitride MXenes. The above results also show that the effect of the MXene thickness on the CO_2_ adsorption nitride MXenes is significantly larger than in carbide MXenes.^[12]^ This is a rather unexpected finding which hinders finding clear systematic trends. However, interesting results are observed when analyzing each case separately. Increasing the thickness markedly increases the CO_2_‐philicity in the Nb‐ and Ta‐derived nitride MXenes, slightly in the Ti‐, Zr‐, Hf‐ and Mo‐derived nitride MXenes, and no effect for the V‐ and Cr‐ ones. The case of nitride W‐MXenes follows an opposite trend as its affinity for CO_2_ decreases by increasing the thickness as further discussed later. Additional interesting insights emerges when comparing to (001) and (111) TMN surfaces. As expected from the trends in surface energy, the TMN (001) surface is clearly less active than the (111) one with a calculated adsorption energy below *ca*. −2.2 eV which is reasonable but not as high as the one corresponding to the TMN (111), behaving effectively as MXene surfaces. This indicates that the chemistry of the hard‐to‐prepare TMN (111) can be more easily studied using the corresponding nitride MXenes. Before closing the discussion regarding adsorption energies, it is worth mentioning that the presence of the CO_2_ on the (001) and (111) CrN, MoN, and WN surfaces promotes superficial deformations inducing reconstruction which has been attributed to the fact that the rock‐salt polymorph of these nitrides is not the most stable one.^[38]^

**Figure 3:**
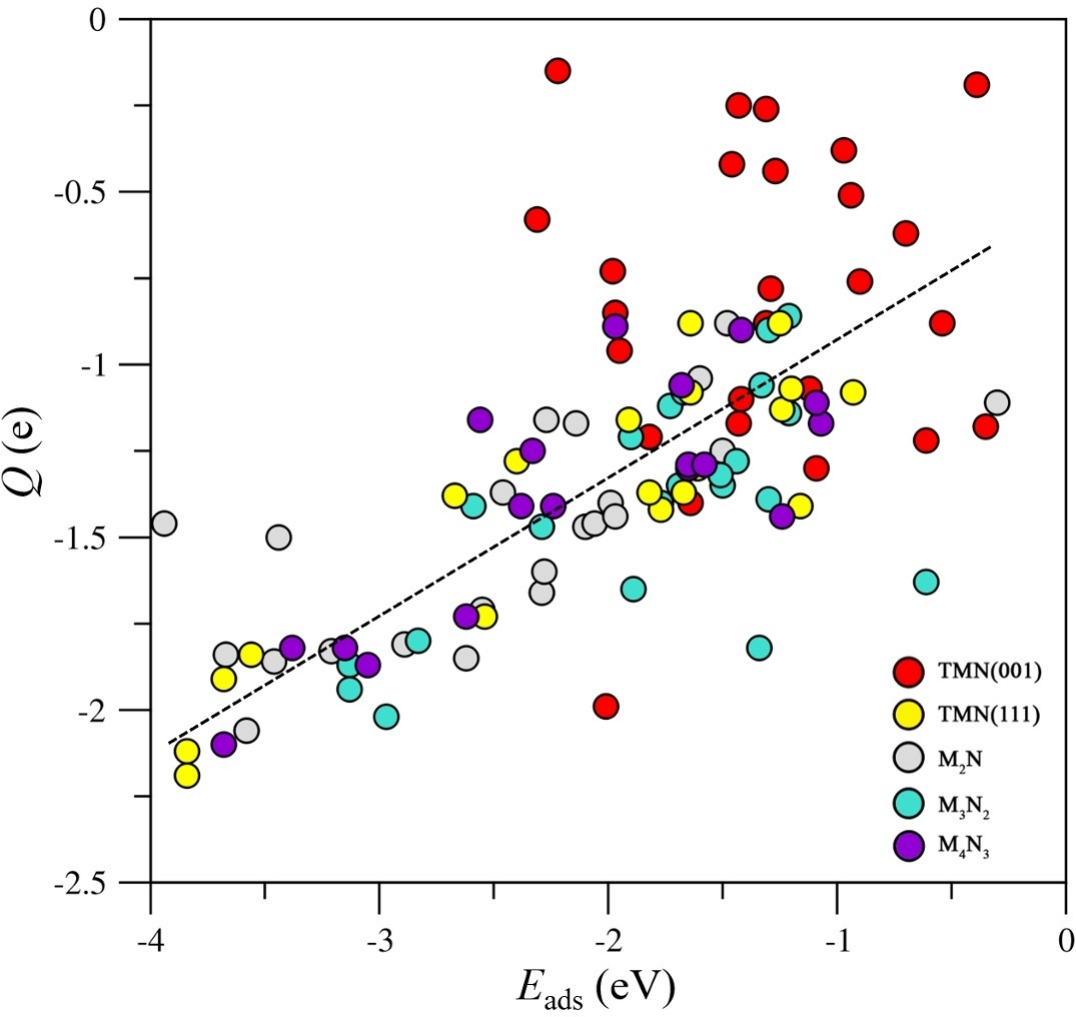
Bader charge on the adsorbed CO_2_ molecule, Q, in *e*, versus adsorption energy, *E*_ads_, in eV, on the MXenes nitrides (M_*n*+1_N_*n*_, *n*=1–3) (0001) surfaces, and TMN (001) and (111) surfaces. Note that all adsorption sites and modes for CO_2_ on the explored surfaces are considered. The dashed black line is a guide view to visualize the trend.

The large adsorption energy of CO_2_ on the (0001) nitride MXene surfaces is accompanied by a considerable charge transfer from the substrate towards CO_2_ which we estimate by adding the net Bader charge in the atoms of the adsorbed molecule. To explore whether a large adsorption energy is coupled to a large charge transfer with a concomitant CO_2_ activation we computed the topological Bader charge, Q, of the CO_2_ molecule and plotted it versus *E*_ads_, see Figure 3. The corresponding plot evidences a clear trend but not a quantitative relationship. It is noteworthy that a similar trend emerges for the TMN (111) surfaces as clearly seen also in Figure 3. This is not surprising because the MXene (0001) and TMN (111) surfaces have a close structural resemblance which translates into a similar behavior towards the CO_2_ adsorption. More in detail, the four set of data show that in the majority of the analyzed substrates, the *E*_ads_ and Q values concentrate in the −2.5 to −1.0 eV and −1.75 to −0.9 *e* range, respectively. The set of TMN (001) surfaces does not follow any clear trend except that low *E*_ads_ values are coupled to small Q on the CO_2_ molecule. This is a clear indication of the relatively stability (low reactivity) of the TMN (001) surfaces. As expected, the charge transfer induces structural changes on the CO_2_ molecule promoting a bending of the O−C−O angle; the activated CO_2_^δ−^ adsorbate having angles in the 111.7–135.6° interval, depending on the substrate. The deviation from linearity is connected to Q but without clear trends. All in all, the results discussed so far support the claim that bare M_*n*+1_N_*n*_ systems are appropriate for CO_2_ abatement.

**Figure 4:**
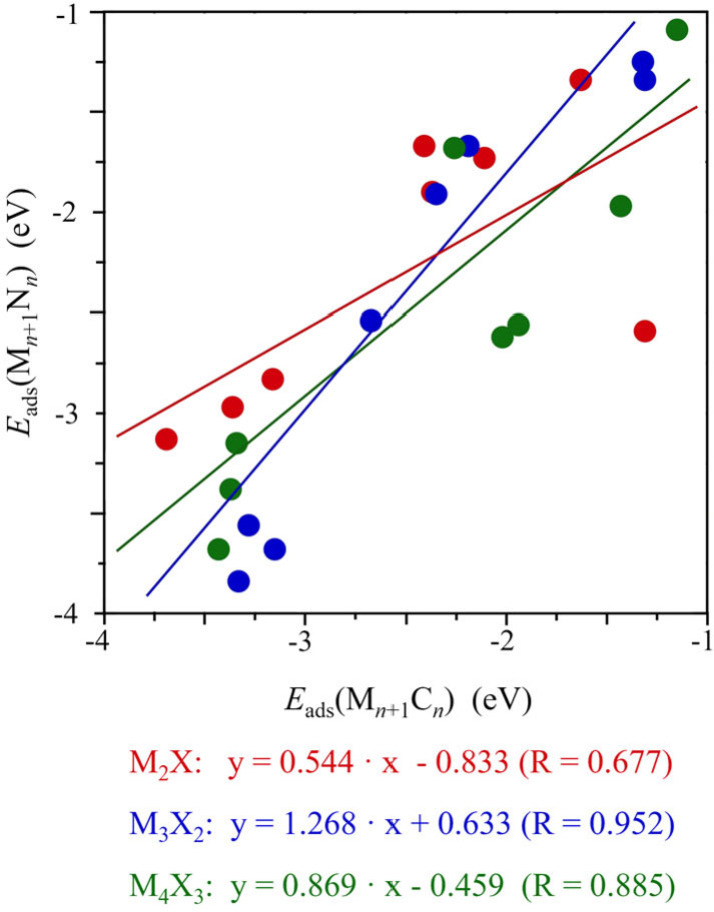
Comparison between *E*_ads_ for nitrides and carbides with different thickness. Red, blue, and green colors correspond to M_2_X, M_3_X_2_, and M_4_X_3_ MXenes. The solid lines stand for the linear fitting. Note that only the most adsorption modes for CO_2_ on the explored surfaces are considered.

At this point, it is interesting to compare the CO_2_ adsorption energy, *E*_ads_, of nitride and carbide MXenes and its role on the activation of CO_2_. We have shown in an earlier work that, in the case of carbide MXenes, the thickness has a small or negligible effect on their interaction with CO_2_.^[12]^ The results above show that thickness effects are much larger for nitride MXenes. To have a deeper insight in the influence of the X element (C or N) on the CO_2_ adsorption, we compare the CO_2_ adsorption energy for the two families of MXenes. To this end, in Figure 4 we plot *E*_ads_ (M_*n*+1_N_*n*_) versus *E*_ads_ (M_*n*+1_C_*n*_) with the data for the nitride MXenes obtained in the present work and that of MXene carbides taken from previous work.^[12]^ The plots in Figure 4, even showing qualitative trends only, evidence that M_2_N and M_4_N_3_ MXenes feature a lower CO_2_‐philicity than their carbide counterparts (M_2_C and M_4_C_3_). On the other hand, M_3_N_2_ MXenes feature a larger affinity for CO_2_ than their carbide counterpart (M_3_C_2_), although the reasons for these behaviors deserve a more detailed analysis which is out of the scope of the present work. Noting that the dispersion of the results, we observe that it is larger in *E*_ads_ (M_*n*+1_N_*n*_) than in *E*_ads_ (M_*n*+1_C_*n*_) and moving also from *d* ^2^‐ to *d* ^4^‐MXenes which indicates that the non‐metallic element in the MXenes plays a non‐negligible role in the chemistry of these materials. According to the present results, one can conclude that the adsorption and activation of CO_2_ could be modulated somehow playing with the nature (carbide or nitride) and the thickness of MXenes which opens a window to optimize pressure and temperature to control the adsorption/desorption process which is very relevant for the possible use of these materials in CCS technologies. This is investigated in detail in the next section.

### 2.2 Pressure and Temperature Effects

Here we describe the results obtained from TST derived adsorption/desorption rates and the resulting kinetic phase diagrams. These display the pressure/temperature regions where adsorption or desorption prevails with the dashed lines indicating the situations where the two rates coincide leading to a dynamic equilibrium situation. Note that these are in general different from the phase diagrams that may be obtained from atomistic thermodynamics^[39]^ which require that the system reaches thermodynamic equilibrium. In the case that thermodynamics equilibrium occurs very rapidly both approaches coincide. Here we prefer to rely on the kinetic phase diagrams because it properly considers the dynamic nature of the equilibrium and the fact that desorption rates may be slow.

**Figure 5:**
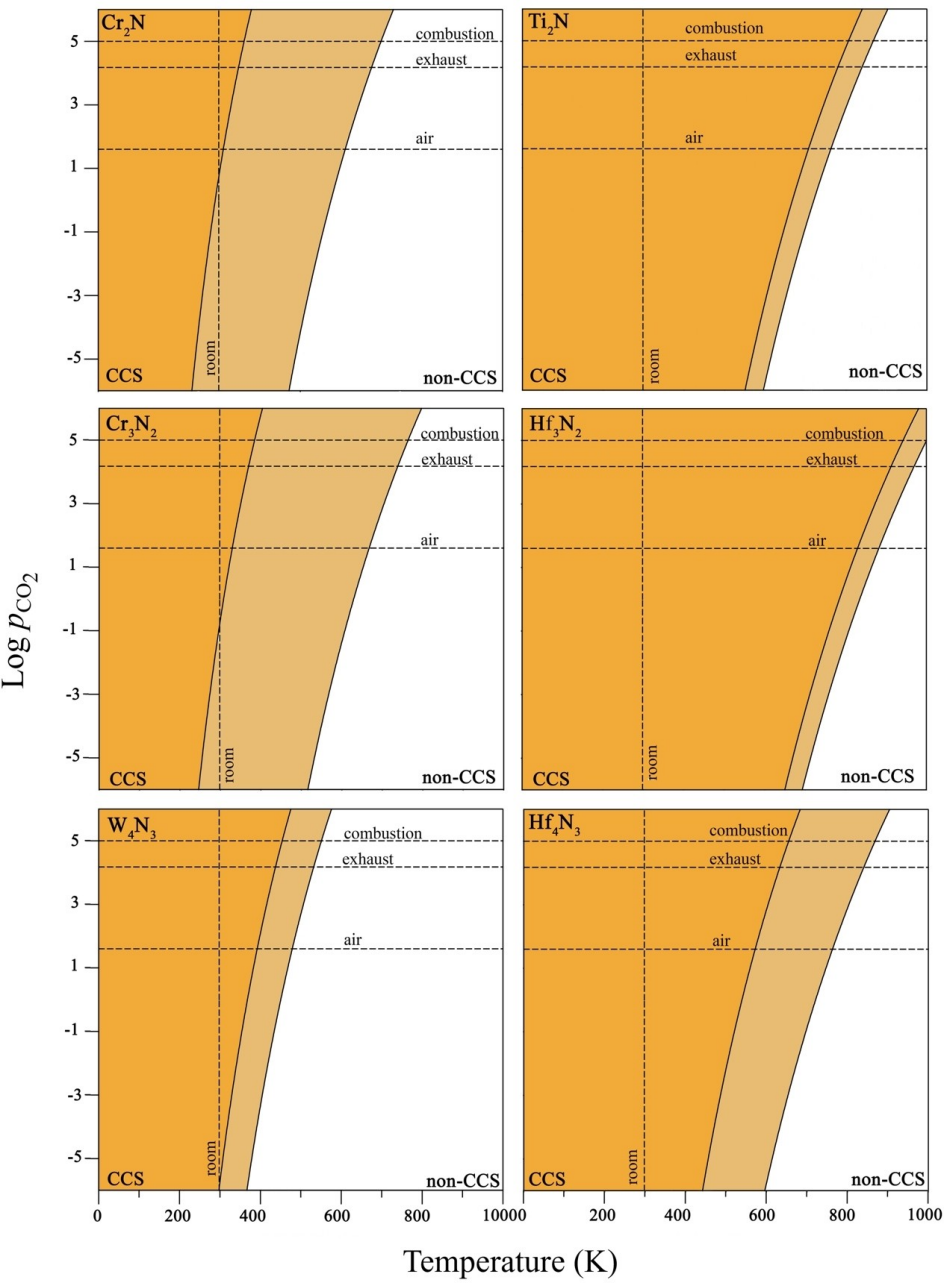
Kinetic phase diagrams for CO_2_ adsorption/desorption on the (0001) M_*n*+1_N_*n*_ MXene surfaces. The crossover among the dashed lines indicates the conditions of CO_2_ partial pressure (*e. g*., air: 40 Pa, exhaust: 15 kPa, and combustion:100 kPa) and room temperature (300 K). The color scheme defines three plausible scenarios CCS, non‐CCS and an intermediate situation that are depicted with dark yellow, white, and light yellow, respectively. Noting that the left and right panels stand for the least and most favorable cases for each MXene stoichiometries and the rest of cases are in the middle and are reported in the SI.

Figure 5 shows the most representative kinetic phase diagrams of bare nitride MXenes with M_2_N, M_3_N_2_, and M_4_N_3_ stoichiometries. We focus mainly on nitride MXenes with the lowest (Cr_2_N, Cr_3_N_2_, and W_4_N_3_) and largest (Ti_2_N, Hf_3_N_2_, and Hf_4_N_3_) CO_2_ adsorption energy, whereas the rest of cases including the TMN surfaces, with situations between the extreme situations shown in Figure 5, are reported in the SI. All kinetic phase diagrams display three well differentiate regions: *i*) one dominated by adsorption and relevant to CCS (dark orange) located at low *T*, *ii*) a transition zone where adsorption and desorption compete (light orange), and finally *iii*) the desorption dominated zone (white), where CO_2_ does not adsorb at all (non‐CCS zone). To provide error bars to the predictions, the border between the transition zones corresponds to the least favorable *E*_ads_ values as predicted by the PBE functional, whereas the border between the CCS transition and the non‐CCS zones corresponds the *E*_ads_ values including dispersion through the PBE‐D3 functional. This defines the light orange region which varies with the composition. Those substrates with a large variety of active sites with similar energetics expose a broader region than those ones whose adsorption energies are similar on different active sites. It must be noted that these limits are conservative because they include all plausible adsorption sites that are placed somewhere between them.

The CCS capability of a given surface depends on the CO_2_ partial pressure, $p_{CO_{2}}$ , and on the temperature. Thus, three actionable $p_{CO_{2}}$ conditions are considered in the present study: *i*) atmospheric pressure where $p_{CO_{2}}$ =40 Pa,^[40]^ *ii*) a benchmark value corresponding to postcombustion exhaust gases and, hence, $p_{CO_{2}}$ =15 kPa,^[41]^ and *iii*) pure CO_2_ stream generation from a CCS system with $p_{CO_{2}}$ =100 kPa (1 bar).^[5]^ From a thermodynamic viewpoint it is convenient that CO_2_ activation occurs at room temperature (*ca*. 300 K). These conditions are essential to achieve efficient CO_2_ capture from air. Indeed, this is the main idea behind the technologies based on the Direct Air Capture (DAC).^[42]^ From the results summarized in Figure 5, the Cr_2_N, Cr_3_N_2_, and W_4_N_3_ MXene (0001) exposed surfaces exhibit the lowest CO_2_‐philicity. Although all partial $p_{CO_{2}}$ at 300 K are located inside the CCS zone, CO_2_ could be easily released just by increasing slightly the temperature at constant pressure. This scenario is especially highlighted on the Cr‐derived MXenes. On the other hand, the Ti_2_N, Hf_3_N_2_, and Hf_4_N_3_ MXene (0001) surfaces featuring the largest *E*_ads_ are appropriate exclusively for CO_2_ trapping, as conditions are well in the CCS region and the shift towards the non‐CCS regime maintaining the $p_{CO_{2}}$ constant requires high temperatures that could compromise somehow the stability of these MXene compounds. Note that the latter MXenes are highly reactive, and their surfaces are passivized by CO_2_ even under mild conditions.

From the preceding discussion Cr_2_N, Cr_3_N_2_, and W_4_N_3_ nitride MXene emerge as promising DAC filters where CO_2_ is chemisorbed but can be easily released. Thus, once the MXene filters become saturated, heating at temperatures close to 500 K release CO_2_ for subsequent use or storage and regenerates the filter. The thus obtained CO_2_ feedstock can be employed in a second step to synthesis of fuels and chemicals based on the heterogeneously catalyzed CO_2_ hydrogenation *via* the reverse water gas shift (RWGS) or Fischer‐Tropsch reactions.^[43]^ Note also that the (001) surfaces of bulk TMNs constitute potential substrates due to their not so high interaction with CO_2_ making feasible the carbon capture and usage.

Before ending this section, we must point out that the present results have been obtained for a low coverage situation and that lateral interactions are likely to decrease the adsorption energy so MXenes with larger *E*_ads_ at low coverage renders CCS suitable while those with smaller *E*_ads_ may end up in being less useful. Note, however, that that the experimental uptake in the work of Persson *et al*.^[15]^ qualitatively agree with predictions for the M_2_C MXenes made using a similar low coverage situation^[11]^ thus indicating that the coverage effects, while important, will not change the overall conclusions of the present work. Besides, the border zone in the kinetic phase diagrams already take into account possible error bars in the adsorption energy.

## 3 Conclusions

The (0001) nitride MXene surfaces with M_*n*+1_N_*n*_ (*n*=1–3; M=Ti, Zr, Hf, V, Nb, Ta, Cr, Mo, and W) stoichiometries and the (001) and (111) TMN surfaces were investigated as plausible substrates for CO_2_ capture. By means of a combination of first‐principles calculations and macroscopic simulations, the role of the thickness and the composition are revealed. The effect of the thickness on CO_2_ adsorption and activation is larger than in the case of carbide MXenes. For a given TM, all nitride MXenes expose similar CO_2_‐philicity but with small, yet noticeable, differences. On the contrary, the effect of the TM is very large, as in carbide MXenes, with Ti, Hf, and Zr‐derived nitride MXenes featuring the most exothermic adsorption followed by V, Nb, and Ta‐derived nitride MXenes and, finally, Cr, Mo, and W‐derived ones. The trends here reported for nitride MXenes of different thickness are somehow different to those reported for carbide MXenes with a much more marked influence of the nitride MXene thickness in the calculated CO_2_ adsorption energy. The analysis of the results also shows that the MXene (0001) surfaces behave as the (111) surface of bulk TMNs. These trends in adsorption energy are consistent with a significant charge transfer from the MXene (or TMN) substrate towards the CO_2_ molecule, a clear indication that, once chemisorbed on the MXenes, the CO_2_ molecule is highly activated which call for additional work to analyze its possible conversion to other useful chemicals. By comparing MXene carbides and nitrides, it is concluded that M_2_N and M_4_N_3_ MXenes have, in general, less affinity for CO_2_ than their carbide counterparts (M_2_C and M_4_C_3_), whereas the family M_3_N_2_ exposes larger affinities than M_3_C_2_. The present results suggest that the CO_2_ adsorption on MXenes can be monitored by selecting the appropriate composition and thickness of the material.

Finally, newly developed kinetic phase diagrams have been introduced that allow one to identify the conditions at which the MXene substrates are able to capture and release CO_2_. These diagrams provide unbiased information that can be useful to experimentalists and engineers working on CCS. We conclude that (Cr, Mo, and W)‐derived MXenes constitute the most suitable materials to act as collector of CO_2_ due to the feasible CO_2_ capture at mild conditions and the affordable release by heating below 500 K. MXene can act as CO_2_ feedstock to heterogenous catalytic processes as the CO_2_ hydrogenation to obtain valuable fuels and chemicals.

## Conflict of interest

The authors declare no conflict of interest.

## Supporting information

As a service to our authors and readers, this journal provides supporting information supplied by the authors. Such materials are peer reviewed and may be re‐organized for online delivery, but are not copy‐edited or typeset. Technical support issues arising from supporting information (other than missing files) should be addressed to the authors.

Supporting Information
