# Supplementary material for: Adsorption and Activation of CO2 on Nitride MXenes: Composition, Temperature, and Pressure effects
Source: Chemphyschem. 2021 Oct 13;22(23):2456–63. doi: 10.1002/cphc.202100600 (PMC9291834; doi:10.1002/cphc.202100600)

ChemPhysChem

Supporting Information

Adsorption and Activation of CO_2 on Nitride MXenes: Composition, Temperature, and Pressure effects

Anabel Jurado, Kevin Ibarra, Ángel Morales-García,* Francesc Viñes, and Francesc Illas

Adsorption and Activation of CO_2 on Nitride MXenes: Composition, Temperature, and Pressure effects

Anabel Jurado, Kevin Ibarra, Ángel Morales-García*, Francesc Viñes, Francesc Illas

Departament de Ciència de Materials i Química Física & Institut de Química Teòrica i Computacional (IQTCUB), Universitat de Barcelona, c/Martí i Franquès 1-11, 08028 Barcelona, Spain

e-mail: angel.morales@ub.edu

Contents

Table S1. Adsorption energy, and structural parameters for the activated CO_2 adsorbed on M_3N_2 MXenes.

Table S2. Adsorption energy, and structural parameters for the activated CO_2 adsorbed on M_4N_3 MXenes.

Table S3. Adsorption energy, and structural parameters for the activated CO_2 adsorbed on Transition Metal Nitrides (TMN) (001) surfaces.

Table S4. Adsorption energy, and structural parameters for the activated CO_2 adsorbed on TMN (111) surfaces.

Fig S1. Top and side views of the CO_2 adsorption sites on the studied TMN (001) surfaces.

Fig S2. Carbon Capture and Storage (CCS) kinetic phase diagrams of Ti-derived MXenes.

Fig S3. CCS kinetic phase diagrams of TiN (001) and (111) surfaces.

Fig S4. CCS kinetic phase diagrams of Zr-derived MXenes.

Fig S5. CCS kinetic phase diagrams of ZrN (001) and (111) surfaces.

Fig S6. CCS kinetic phase diagrams of Hf-derived MXenes.

Fig S7. CCS kinetic phase diagrams of HfN (001) and (111) surfaces.

Fig S8. CCS kinetic phase diagrams of V-derived MXenes.

Fig S9. CCS kinetic phase diagrams of VN (001) and (111) surfaces.

Fig S10. CCS kinetic phase diagrams of Nb-derived MXenes.

Fig S11. CCS kinetic phase diagrams of NbN (001) and (111) surfaces.

Fig S12. CCS kinetic phase diagrams of Ta-derived MXenes.

Fig S13. CCS kinetic phase diagrams of TaN (001) and (111) surfaces.

Fig S14. CCS kinetic phase diagrams of Cr-derived MXenes.

Fig S15. CCS kinetic phase diagrams of CrN (001) and (111) surfaces.

Fig S16. CCS kinetic phase diagrams of Mo-derived MXenes.

Fig S17. CCS kinetic phase diagrams of MoN (001) and (111) surfaces.

Fig S18. CCS kinetic phase diagrams of W-derived MXenes.

Fig S19. CCS kinetic phase diagrams of WN (001) and (111) surfaces.

Table S1. Adsorption energy, E_{ads}, and structural parameters, including CO_2 C-O bond distance, δ(CO), distance between surface metal atoms and CO_2 O atoms, δ(MO), and CO_2 molecular angle, α(OCO), for the activated CO_2 adsorbed on M_3N_2 MXenes on different sites. Q corresponds to the net Bader charge of the activated CO_2. Bolt font indicates the most exothermic E_{ads} for each MXene substrate.

MXene	DFT	E_{ads}/eV	δ(CO)/Å	δ(MO)/Å	α(OCO)/º	Q/*e*
			**C_b**			
Hf_3N_2	PBE	-3.55	1.38/1.43	2.27/2.29	113.4	-2.14
	PBE-D3	-3.84	1.39/1.41	2.28/2.25	114.0	-2.12
V_3N_2	PBE	-1.36	1.26	2.11	136.5	-1.07
	PBE-D3	-1.64	1.27	2.11	134.7	-1.08
Nb_3N_2	PBE	**-1.63**	1.28	2.20	132.3	-1.15
	PBE-D3	**-1.91**	1.28	2.20	132.3	-1.16
Ta_3N_2	PBE	-2.16	1.29	2.12	131.2	-1.28
	PBE-D3	-2.40	1.29	2.12	131.2	-1.28
Cr_3N_2	PBE	**-0.91**	1.26	2.11	136.5	-0.93
	PBE-D3	**-1.58**	1.26	2.08	137.2	-0.88
Mo_3N_2	PBE	**-0.85**	1.26	2.28	135.4	-0.89
	PBE-D3	**-1.25**	1.26	2.26	136.0	-0.88
			**$C_bO_bO_b$**			
Cr_3N_2	PBE	-1.10	1.40/1.29	1.95/2.11	121.7	-1.28
	PBE-D3	-1.43	1.38/1.30	2.06/2.01	119.2	-1.38
Mo_3N_2	PBE	-0.86	1.38/1.36	2.35/2.21	111.7	-1.41
	PBE-D3	-1.16	1.39/1.36	2.24/2.19	111.7	-1.41
			**C_mO_b**			
V_3N_2	PBE	**-1.38**	1.35/1.26	2.20/2.28	132.8	-1.37
	PBE-D3	**-1.67**	1.26/1.34	2.21/2.28	132.9	-1.37
Nb_3N_2	PBE	-1.49	1.27/1.33	2.34/2.36	132.0	-1.42
	PBE-D3	-1.77	1.27/1.33	2.34	132.9	-1.42
Mo_3N_2	PBE	-0.61	1.29/1.26	2.47/2.33	134.4	-1.07
	PBE-D3	-0.93	1.26/1.29	2.46/2.32	134.4	-1.08
			**$C_mO_nO_n$**			
Ti_3N_2	PBE	**-3.30**	1.38	2.20	115.0	-1.85
	PBE-D3	**-3.56**	1.38	2.20	115.1	-1.84
Zr_3N_2	PBE	**-3.42**	1.38/1.39	2.33	114.9	-1.90
	PBE-D3	**-3.68**	1.38/1.39	2.33	114.8	-1.91
Hf_3N_2	PBE	**-3.55**	1.37/1.45	2.31/2.28	113.1	-2.19
	PBE-D3	**-3.84**	1.37/1.44	2.30	113.2	-2.19
			**C_nO_b**			
V_3N_2	PBE	-1.31	1.32/1.26	2.21/2.23	134.1	-1.3
	PBE-D3	-1.61	1.32/1.26	2.22	134.2	-1.3
Nb_3N_2	PBE	-1.54	1.28	2.20	131.3	-1.37
	PBE-D3	-1.82	1.33/1.27	2.33	131.7	-1.37
Cr_3N_2	PBE	**-1.20**	1.26/1.32	2.20/2.18	134.2	-1.20
	PBE-D3	**-1.52**	1.26/1.30	2.17/2.19	135.6	-1.13
			**$C_nO_mO_m$**			

Ta_3N_2	PBE	**-2.32**	1.40	1.98/1.95	111.1	-1.73
	PBE-D3	**-2.54**	1.40	1.98/1.96	111.1	-1.73
			**$C_mO_mO_b$**			
W_3N_2	PBE	**-1.05**	1.32/1.26	2.40	123.6	-1.07
	PBE-D3	**-1.34**	1.34/1.26	2.40	122.6	-1.07

Tabla S2. Adsorption energy, E_{ads}, and structural parameters, see Table S1, for the activated CO_2 adsorbed on M_4N_3 MXenes. Q corresponds to the net Bader charge of the activated CO_2. Bolt font indicates the most exothermic E_{ads} for each MXene substrate.

MXene	DFT	E_{ads}/eV	δ(CO)/Å	δ(MO)/Å	α(OCO)/º	Q/*e*
			**C_b**			
V_4N_3	PBE	**-1.38**	1.27	2.10	135.5	-1.08
	PBE-D3	**-1.68**	1.27	2.10	135.5	-1.06
Nb_4N_3	PBE	**-2.28**	1.28	2.17	131.7	-1.15
	PBE-D3	**-2.56**	1.28	2.17	135.7	-1.16
Ta_4N_3	PBE	-2.02	1.29	2.12	131.8	-1.24
	PBE-D3	-2.33	1.29	2.12	131.7	-1.25
Cr_4N_3	PBE	-1.08	1.26	2.08/2.10	137.2	-0.89
	PBE-D3	-1.42	1.26	2.07/2.09	137.3	-0.90
Mo_4N_3	PBE	-1.26	1.25/1.28	2.28	136.2	-0.87
	PBE-D3	-1.97	1.26	2.26	135.8	-0.89
			**$C_bO_bO_b$**			
Cr_4N_3	PBE	**-1.29**	1.29/1.39	2.10/1.96	121.3	-1.30
	PBE-D3	**-1.65**	1.30/1.39	2.08/1.98	121.7	-1.30
Mo_4N_3	PBE	-1.12	1.36/1.39	2.27/2.30	111.5	-1.43
	PBE-D3	-1.24	1.37/1.39	2.24/2.31	111.4	-1.44
			**C_mO_b**			
V_4N_3	PBE	-1.35	1.32/1.26	2.20/2.25	134.8	-1.30
	PBE-D3	-1.65	1.32/1.26	2.21/2.24	134.8	-1.29
Nb_4N_3	PBE	-1.95	1.34/1.25	2.30/2.40	132.7	-1.41
	PBE-D3	-2.24	1.34/1.27	2.30/2.40	132.7	-1.41
			**$C_mO_nO_n$**			
Ti_4N_3	PBE	**-3.07**	1.37	2.22	115.8	-1.82
	PBE-D3	**-3.38**	1.38	2.22	115.8	-1.82
Zr_4N_3	PBE	**-2.89**	1.37	2.34	115.4	-1.83
	PBE-D3	**-3.15**	1.37	2.34	115.5	-1.82
Hf_4N_3	PBE	**-3.42**	1.36/1.43	2.31/2.22	113.4	-2.11
	PBE-D3	**-3.68**	1.37/1.42	2.30	113.7	-2.10
			**C_nO_b**			
V_4N_3	PBE	-1.28	1.32/1.26	2.21	134.8	-1.28
	PBE-D3	-1.58	1.32/1.26	2.24/2.21	134.8	-1.29
Nb_4N_3	PBE	-2.07	1.32/1.29	2.37/2.22	131.9	-1.40
	PBE-D3	-2.38	1.32/1.29	2.37/2.23	131.8	-1.41
Cr_4N_3	PBE	-0.89	1.25/1.31	2.15/2.22	134.9	-1.13
	PBE-D3	-1.07	1.25/1.32	1.25/1.32	131.7	-1.17
			**$C_nO_mO_m$**			
Ti_4N_3	PBE	-2.74	1.34/1.46	2.29/2.11	114.2	-1.87
	PBE-D3	-3.05	1.34/1.46	2.29/2.14	114.1	-1.87
Ta_4N_3	PBE	**-2.37**	1.40	1.96	111.7	-1.75
	PBE-D3	**-2.62**	1.40	1.96	111.9	-1.73
			**$C_mO_mO_b$**			
W_4N_3	PBE	**-0.84**	1.24/1.41	2.26/2.34	125.7	-1.11

PBE-D3	**-1.09**	1.22/1.5	2.29/2.35	124.9	-1.11

Tabla S3. Adsorption energy, E_{ads}, and structural parameters, see Table S1, for the activated CO_2 adsorbed on Transition Metal Nitrides (TMN) (001) surfaces. Q corresponds to the net Bader charge of the activated CO_2. Bolt font indicates the most exothermic E_{ads} for each MXene substrate.

MXene	DFT	E_{ads}/eV	δ(CO)/Å	δ(MO)/Å	α(OCO)/º	Q/e
			**C_b**			
TiN	PBE	-0.27	1.26	2.22	137.2	-0.89
	PBE-D3	-0.54	1.25	2.22	137.5	-0.88
ZrN	PBE	-1.2	1.27	2.33	131.6	-1.10
	PBE-D3	-1.42	1.27	2.33	131.6	-1.10
HfN	PBE	**-1.57**	1.28	2.27	131.5	-1.21
	PBE-D3	**-1.82**	1.28	2.27	131.6	-1.21
TaN	PBE	-1.09	1.26	2.26	135.4	-0.97
	PBE-D3	-1.12	1.24	2.20	131.9	-1.07
			Top			
TiN	PBE	**-0.68**	1.28	2.25	128.9	-0.38
	PBE-D3	**-0.97**	1.29	2.25	129.0	-0.38
ZrN	PBE	**-1.21**	1.3	2.34	125.8	-1.98
	PBE-D3	**-2.01**	1.3	2.33	125.7	-1.99
HfN	PBE	-1.12	1.3	2.29	127.1	-0.88
	PBE-D3	-1.31	1.3	2.29	127.2	-0.88
VN	PBE	-0.27	1.28	2.25	130.3	-1.22
	PBE-D3	-0.61	1.28	2.25	130.4	-1.22
NbN	PBE	-0.97	1.29	2.37	128.9	-0.26
	PBE-D3	-1.43	1.29	2.36	128.9	-0.25
TaN	PBE	-0.84	1.29	2.27	129.1	-0.45
	PBE-D3	-1.27	1.29	2.3	130.3	-0.44
CrN	PBE	-1.54	1.29	2.15	130.7	-0.95
	PBE-D3	-1.95	1.28	2.16	132	-0.96
MoN	PBE	-0.12	1.27	2.28	130.6	-0.20
	PBE-D3	-0.39	1.22/1.33	2.12/3.70	129.8	-0.19
WN	PBE	-1.37	1.31	2.13	120.7	-0.84
	PBE-D3	-1.97	1.31	2.18	119.4	-0.85
			MMC			
TiN	PBE	-0.62	1.30	2.11	123.6	-0.64
	PBE-D3	-0.90	1.29	2.11	123.7	-0.76
ZrN	PBE	-1.05	1.30	2.25	125.8	-0.78
	PBE-D3	-1.29	1.30	2.25	122.8	-0.78
HfN	PBE	-1.12	1.33	2.13	120.2	-0.27
	PBE-D3	-1.31	1.33	2.13	120.6	-0.26
VN	PBE	**-0.33**	1.29	2.23	124.9	-0.46
	PBE-D3	**-0.70**	1.29	2.80	124.3	-0.62
NbN	PBE	**-1.12**	1.30	2.23	124.9	-0.34
	PBE-D3	**-1.46**	1.30	2.23	127.8	-0.42
TaN	PBE	**-1.94**	1.32	2.09	117.3	-0.53
	PBE-D3	**-2.31**	1.32	2.09	117.2	-0.58
CrN	PBE	**-1.79**	1.29	2.09	126.5	-0.06
	PBE-D3	**-2.22**	1.29	2.8	126.6	-0.15

MoN	PBE	**-0.92**	1.32	2.13	120	-0.34
	PBE-D3	**-1.22**	1.32	2.13	119.9	-0.51
WN	PBE	**-1.38**	1.35	2.14	124.2	-0.66
	PBE-D3	**-1.98**	1.38	2.08	123.9	-0.73
			O_mO_m			
TiN	PBE	-0.01	1.29	2.05	121.3	-1.1
	PBE-D3	-0.35	1.28	2.06	122.9	-1.8
ZrN	PBE	-0.87	1.31	2.12	114.9	-1.29
	PBE-D3	-1.09	1.31	2.13	115.3	-1.3
HfN	PBE	-1.41	1.33	2.06	115.3	-1.4
	PBE-D3	-1.64	1.33	2.06	112.9	-1.4
TaN	PBE	-1.12	1.31	2.09	118.9	-1.17
	PBE-D3	-1.43	1.31	2.04	114.72	-1.17

Tabla S4. Adsorption energy, E_{ads}, and structural parameters, see Table S1, for the activated CO_2 adsorbed on TMN (111) surfaces. Q corresponds to the net Bader charge of the activated CO_2. Bolt font indicates the most exothermic E_{ads} for each MXene substrate.

MXene	Level	Eads/eV	δ(CO)/Å	δ(MO)/Å	α(OCO)/º	Q/e
			**C_b**			
VN	PBE	-1.3	1.27	2.11	135.8	-1.04
	PBE-D3	-1.6	1.27	2.10	135.7	-1.04
NbN	PBE	**-1.86**	1.29	2.19	129.9	-1.16
	PBE-D3	**-2.14**	1.29	2.19	129.9	-1.17
TaN	PBE	-2.28	1.30	2.12	129.1	-1.37
	PBE-D3	-2.46	1.29	2.12	131.7	-1.37
CrN	PBE	-1.04	1.26	2.09/2.11	137.3	-0.87
	PBE-D3	-1.48	1.26	2.08/2.09	137.3	-0.88
MoN	PBE	**-3.27**	1.37	2.20	118.7	-1.49
	PBE-D3	**-3.44**	1.27	2.27	133.3	-0.89
			**$C_bO_bO_b$**			
VN	PBE	-1.68	1.34	1.95	116.6	-1.39
	PBE-D3	-1.99	1.34	1.95	116.6	-1.4
TaN	PBE	-2.18	1.37/1.42	2.11/2.22	106.4	-1.72
	PBE-D3	-2.29	1.35/1.43	2.09/2.18	106.7	-1.66
CrN	PBE	-1.25	1.30/1.40	1.98/2.10/2.21	120.0	-1.33
	PBE-D3	-1.52	1.35	1.93	116.9	-1.29
			**C_mO_b**			
VN	PBE	**-1.83**	1.35	1.95	115.1	-1.47
	PBE-D3	**-2.10**	1.35	1.95	115.1	-1.47
NbN	PBE	-1.47	1.35	2.05	116.5	-1.47
	PBE-D3	-2.06	1.34	2.06	116.5	-1.46
			**$C_mO_nO_n$**			
TiN	PBE	**-3.35**	1.38	2.19/2.22	115.6	-1.83
	PBE-D3	**-3.67**	1.38	2.19/2.22	115.6	-1.84
ZrN	PBE	**-3.20**	1.38	2.34	115.2	-1.85
	PBE-D3	**-3.46**	1.38	2.34	115.1	-1.86
HfN	PBE	**-3.28**	1.39	2.29	113.9	-2.06
	PBE-D3	**-3.58**	1.40	2.29	113.9	-2.06
			**C_nO_b**			
ZrN	PBE	-2.21	1.29/1.36	2.38	130.1	-1.63
	PBE-D3	-2.28	1.29/1.36	2.32/2.37	130.1	-1.6
HfN	PBE	-2.35	1.30/1.39	2.28/2.31	130.5	-1.83
	PBE-D3	-2.62	1.29/1.41	2.3	130.4	-1.85
VN	PBE	-1.2	1.26/1.32	2.19/2.28	134.6	-1.25
	PBE-D3	-1.5	1.26/1.32	2.19/2.24	134.7	-1.25
NbN	PBE	-1.71	1.28/1.33	2.27/2.40	131.5	-1.39
	PBE-D3	-1.97	1.30/1.33	2.26/2.39	129.3	-1.44
CrN	PBE	-0.88	1.26/1.31	2.19/2.23	134.9	-1.15
	PBE-D3	-1.04	1.28	2.0/2.13	125.1	-1.16
			**$C_nO_mO_m$**			
TiN	PBE	-2.97	1.40	2.15/2.23	116.0	-1.85

	PBE-D3	-3.21	1.38	2.19/2.22	115.7	-1.83
ZrN	PBE	-2.64	1.33/1.42	2.29/2.41	115.9	-1.81
	PBE-D3	-2.89	1.33/1.42	2.29/2.40	115.9	-1.81
TaN	PBE	**-2.36**	1.40	1.96	111.8	-1.72
	PBE-D3	**-2.55**	1.40	1.97	111.9	-1.71
CrN	PBE	**-2.99**	1.33/1.36	1.85/1.97	119.4	-1.23
	PBE-D3	**-3.94**	1.33/1.37	2.06/2.17	120.9	-1.46
			**$C_mO_mO_b$**			
WN	PBE	**-0.25**	1.23/1.41	2.27/2.33	125.8	-1.00
	PBE-D3	**-0.39**	1.22/1.50	2.27/2.33	125.0	-1.11

Fig S1. Top and side views of the CO_2 adsorption sites on the studied TMN (001) surfaces as listed in Table S3.

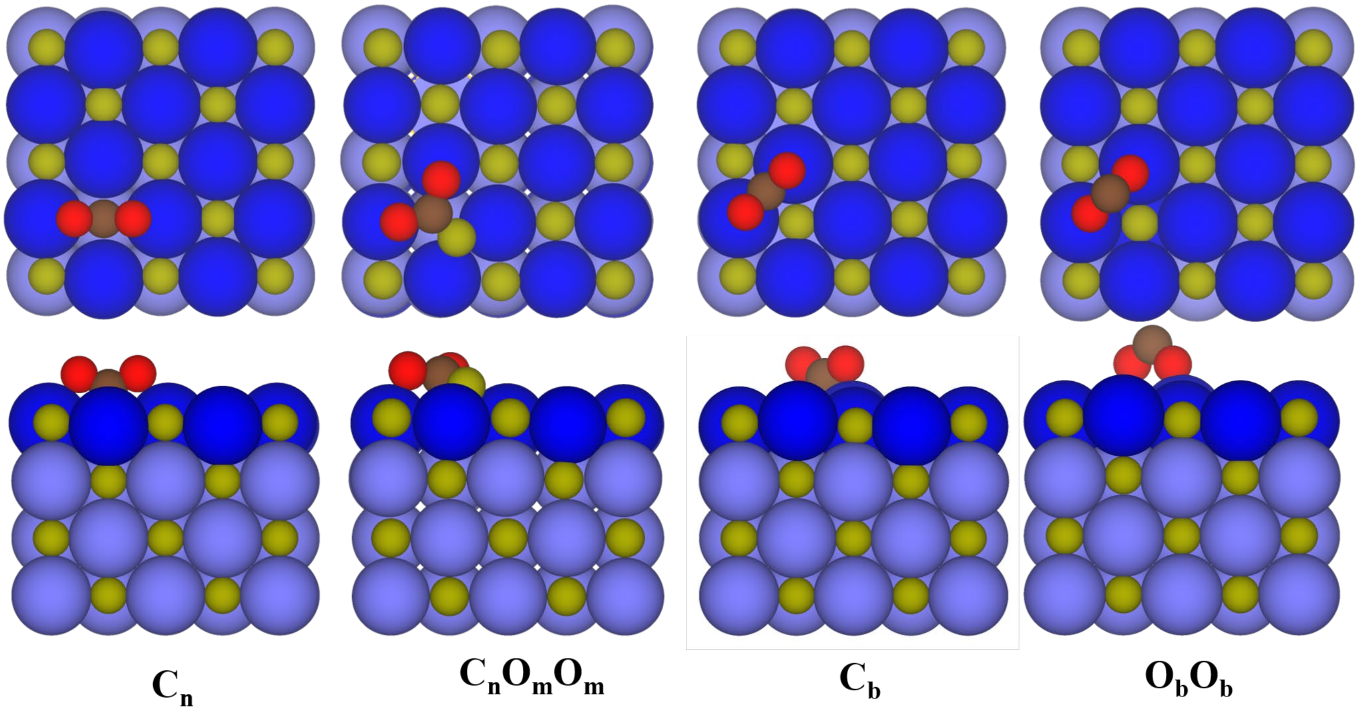

Fig S2. Carbon Capture and Storage (CCS) kinetic phase diagrams of Ti-derived MXenes with stoichiometries Ti_2N, Ti_3N_2, and Ti_4N_3.

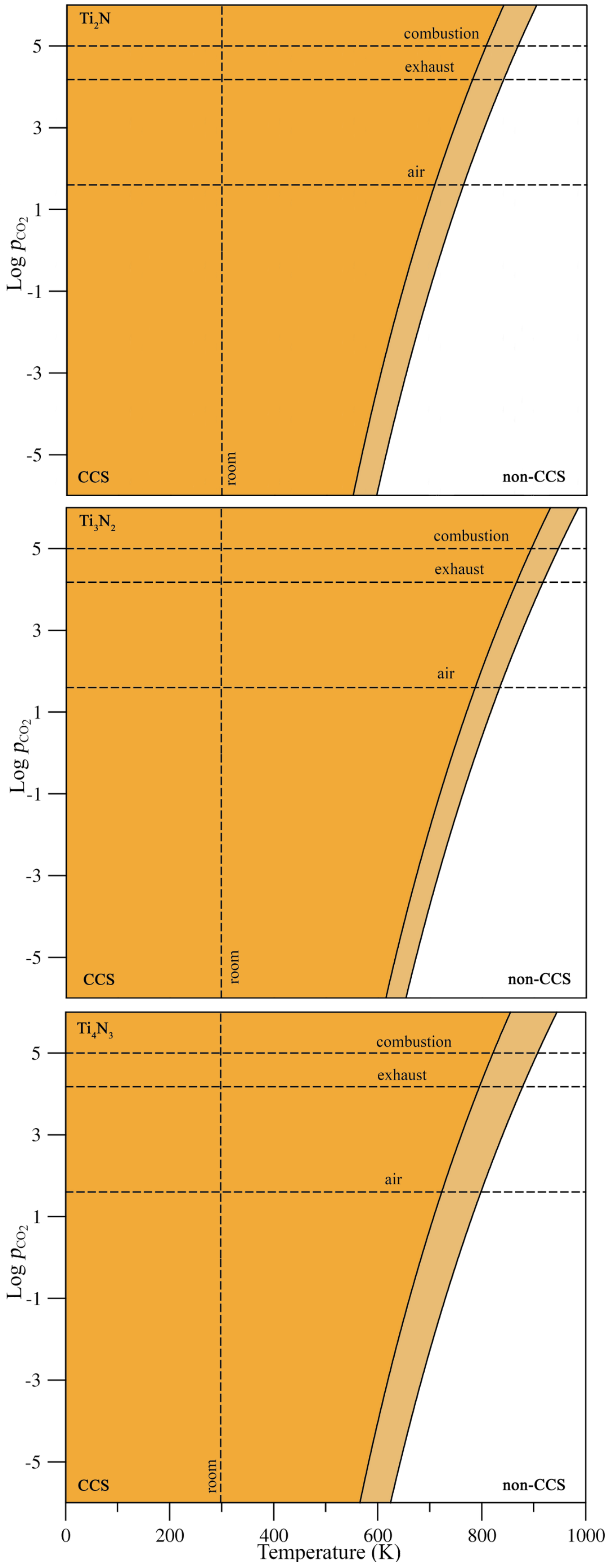

Fig S3. CCS kinetic phase diagrams of TiN (001) and (111) surfaces.

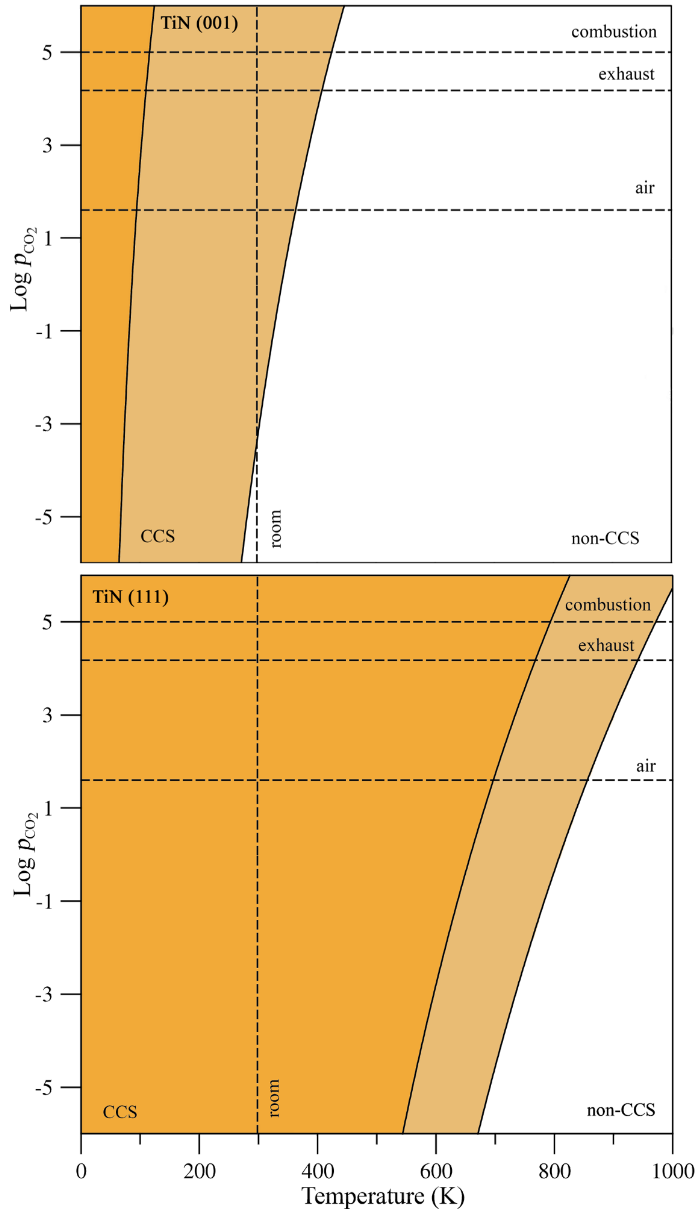

Fig S4. CCS kinetic phase diagrams of CCS) kinetic phase diagrams of Zr-derived MXenes with stoichiometries Zr_2N, Zr_3N_2, and Zr_4N_3.

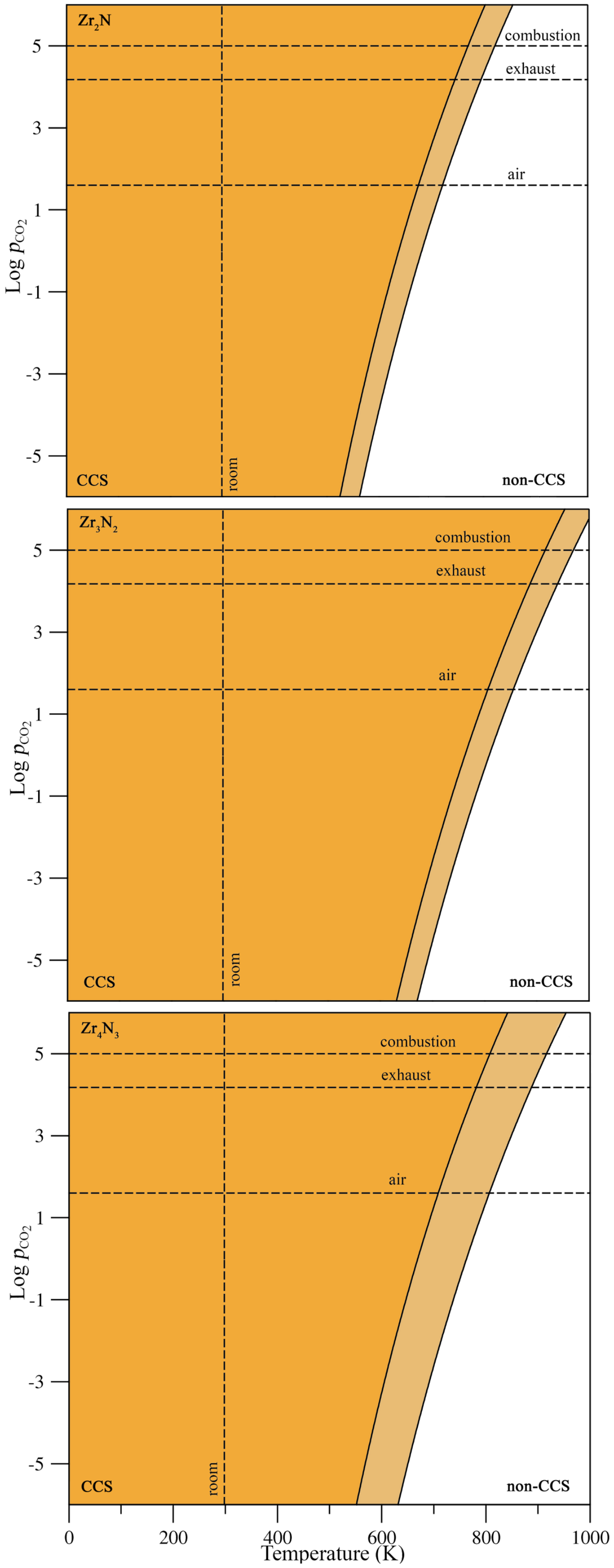

Fig S5. CCS kinetic phase diagrams of ZrN (001) and (111) surfaces.

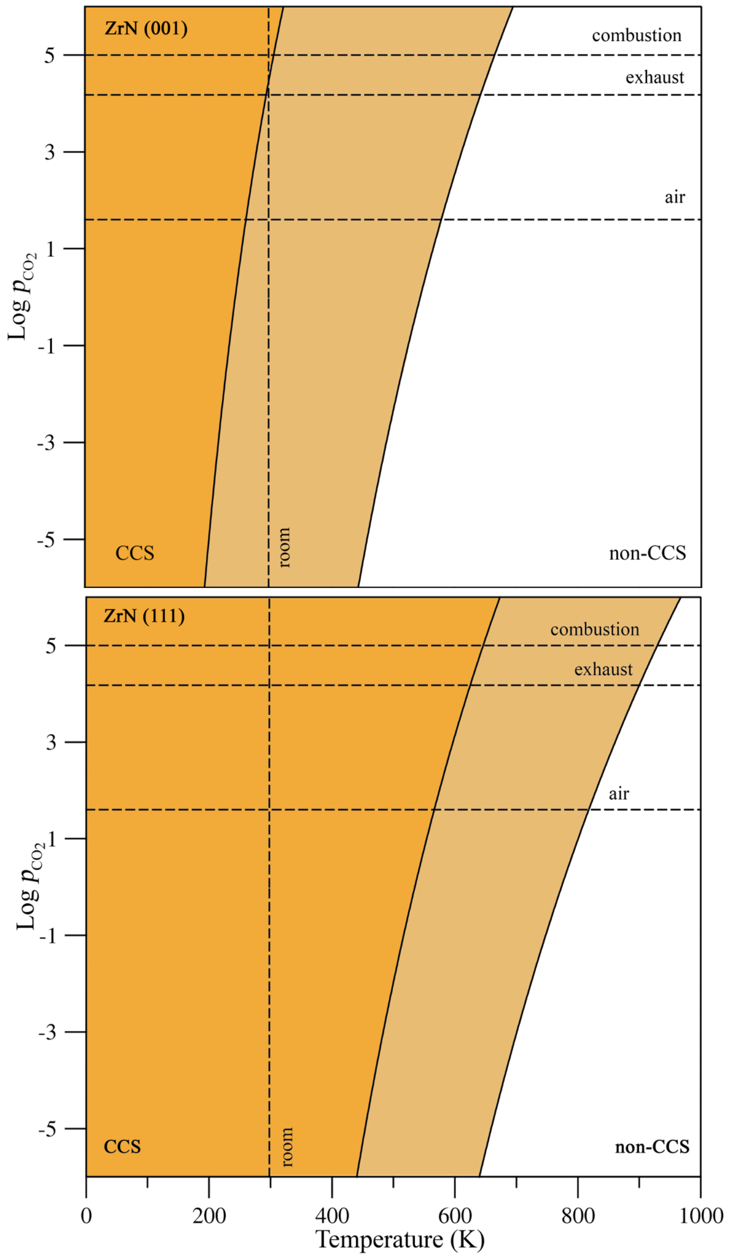

Fig S6. CCS kinetic phase diagrams of CCS) kinetic phase diagrams of Hf-derived MXenes with stoichiometries Hf_2N, Hf_3N_2, and Hf_4N_3.

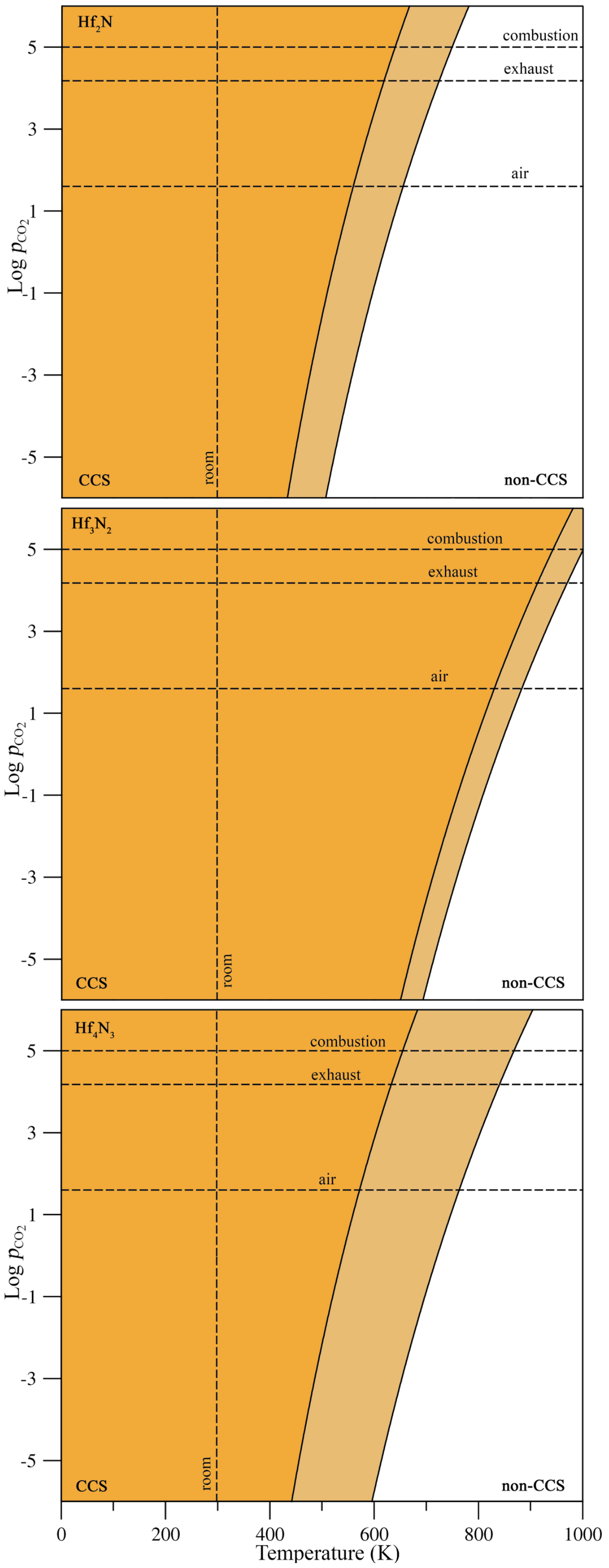

Fig S7. CCS kinetic phase diagrams of HfN (001) and (111) surfaces.

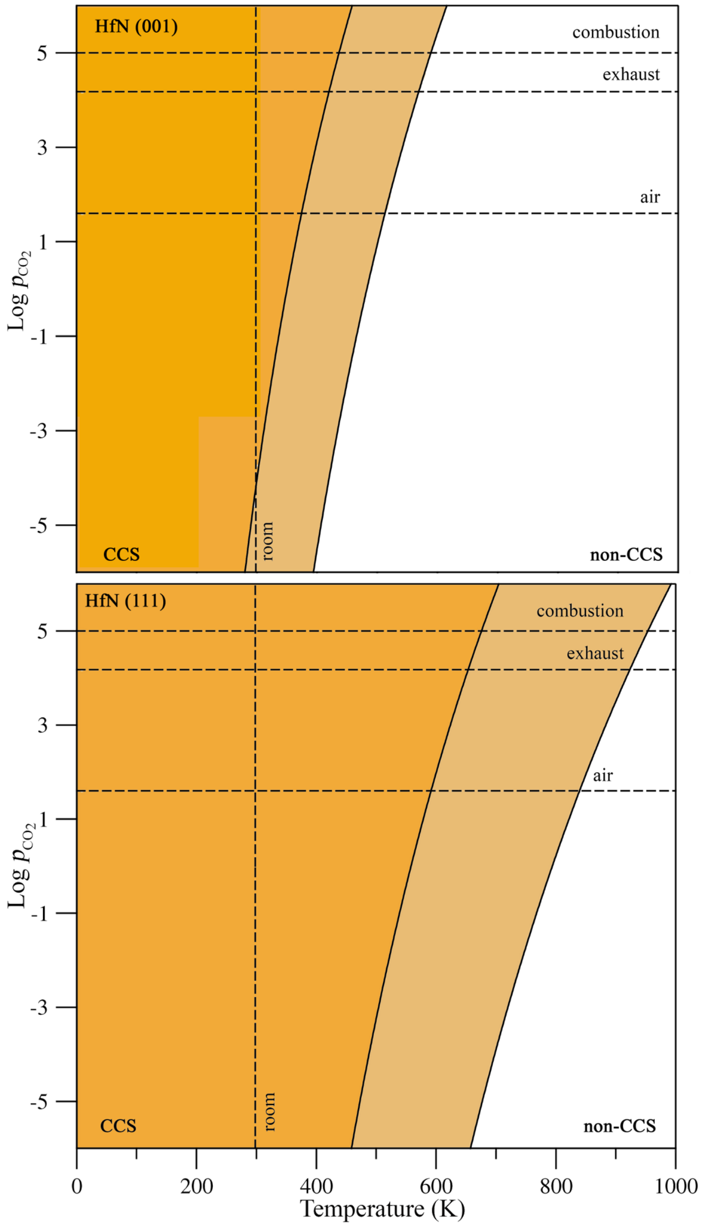

Fig S8. CCS kinetic phase diagrams of CCS) kinetic phase diagrams of V-derived MXenes with stoichiometries V_2N, V_3N_2, and V_4N_3.

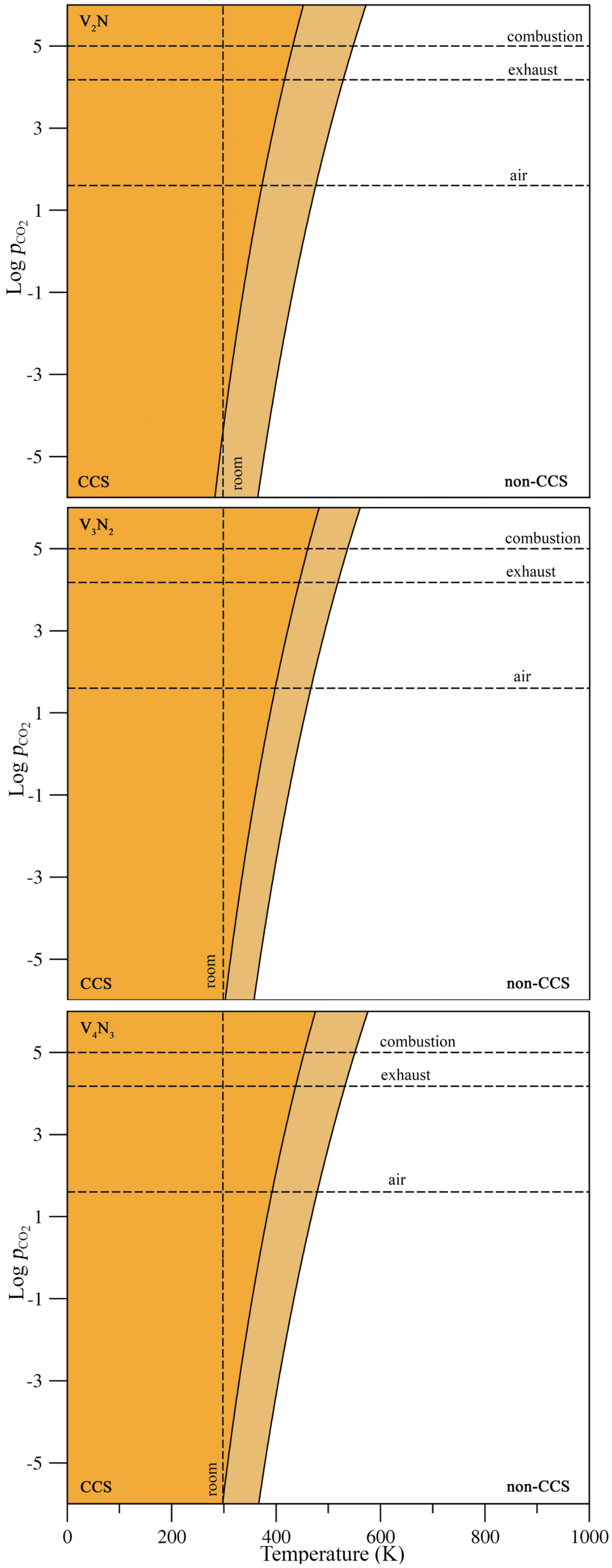

Fig S9. CCS kinetic phase diagrams of VN (001) and (111) surfaces.

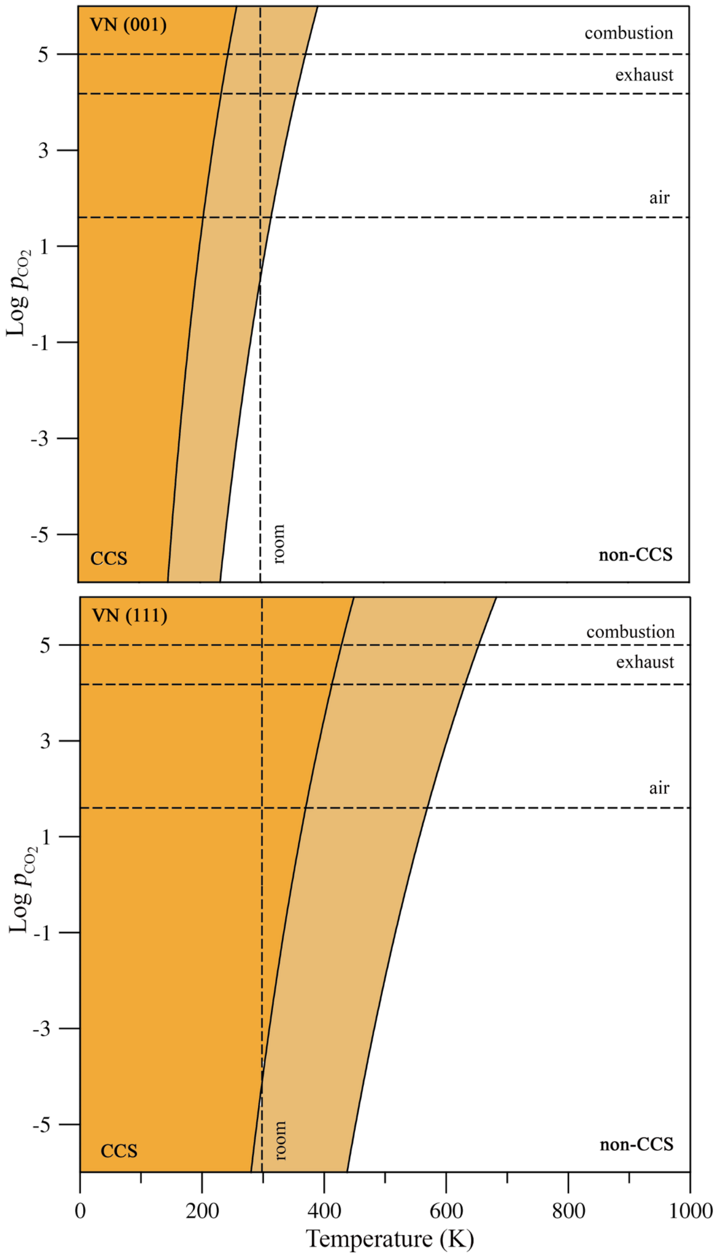

Fig S10. CCS kinetic phase diagrams of CCS) kinetic phase diagrams of Nb-derived MXenes with stoichiometries Nb_2N, Nb_3N_2, and Nb_4N_3.

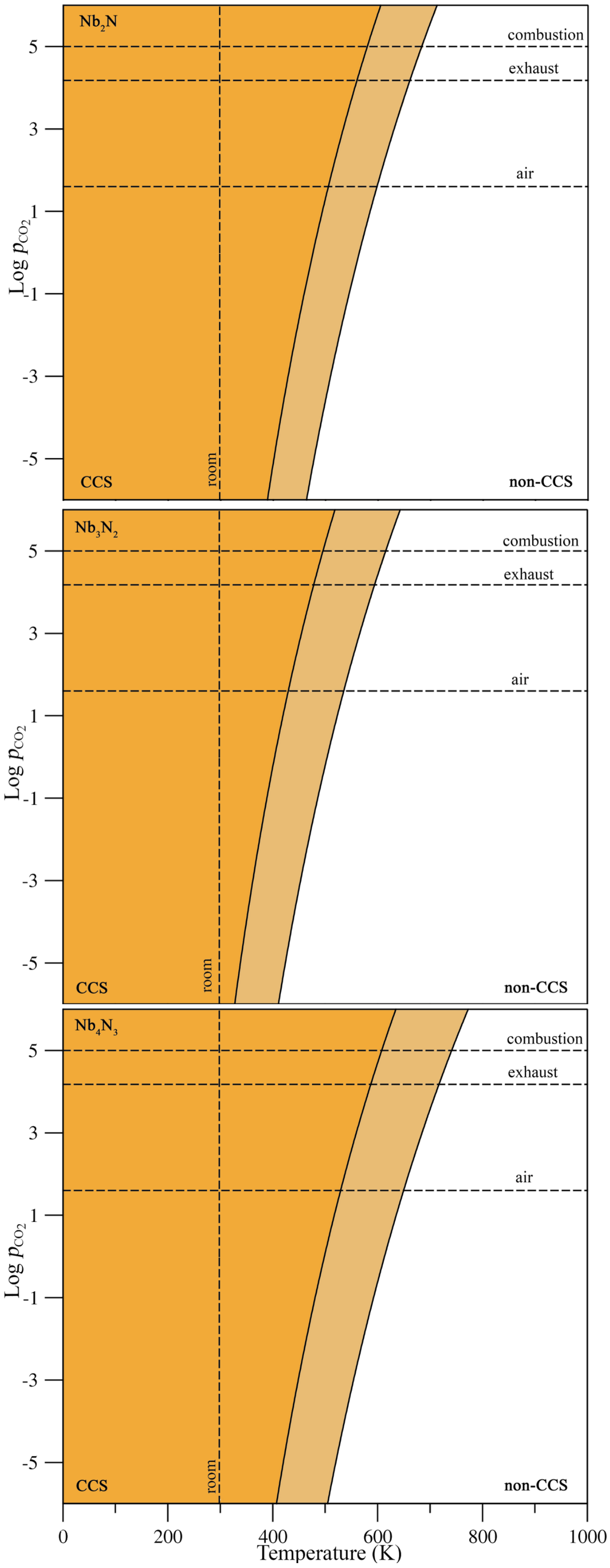

Fig S11. CCS kinetic phase diagrams of NbN (001) and (111) surfaces.

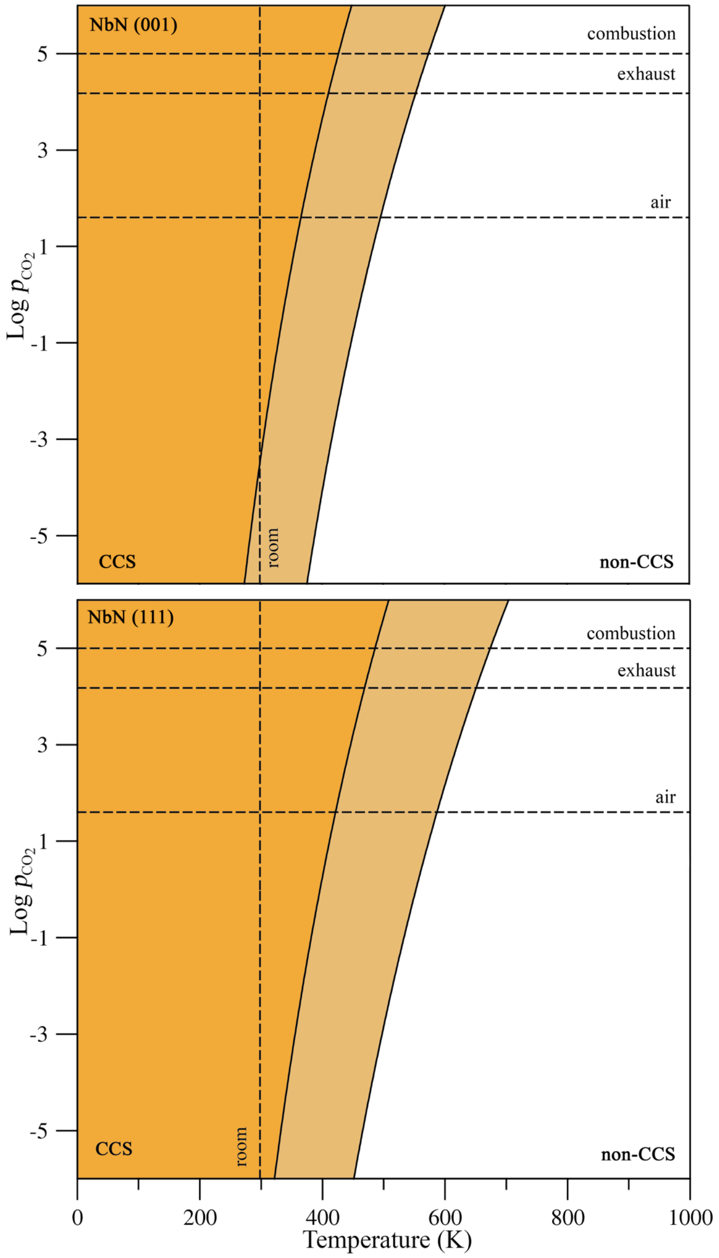

Fig S12. CCS kinetic phase diagrams of CCS) kinetic phase diagrams of Ta-derived MXenes with stoichiometries Ta_2N, Ta_3N_2, and Ta_4N_3.

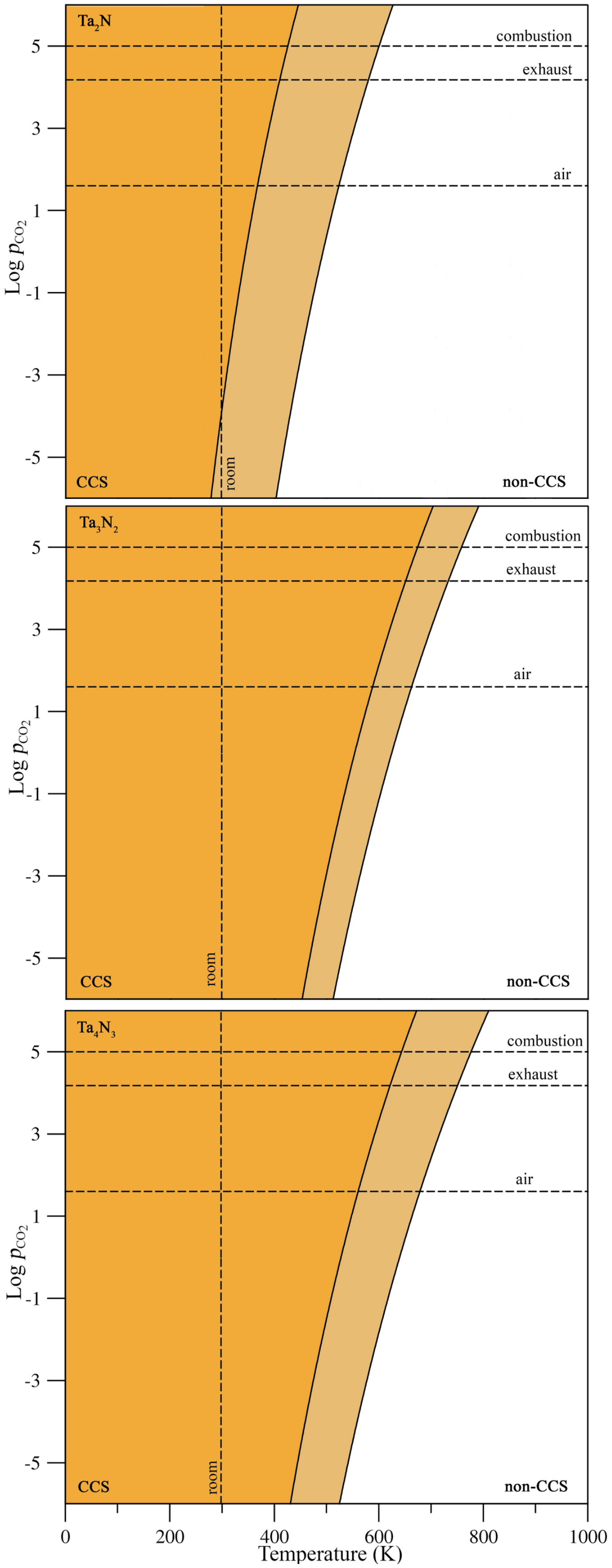

Fig S13. CCS kinetic phase diagrams of TaN (001) and (111) surfaces.

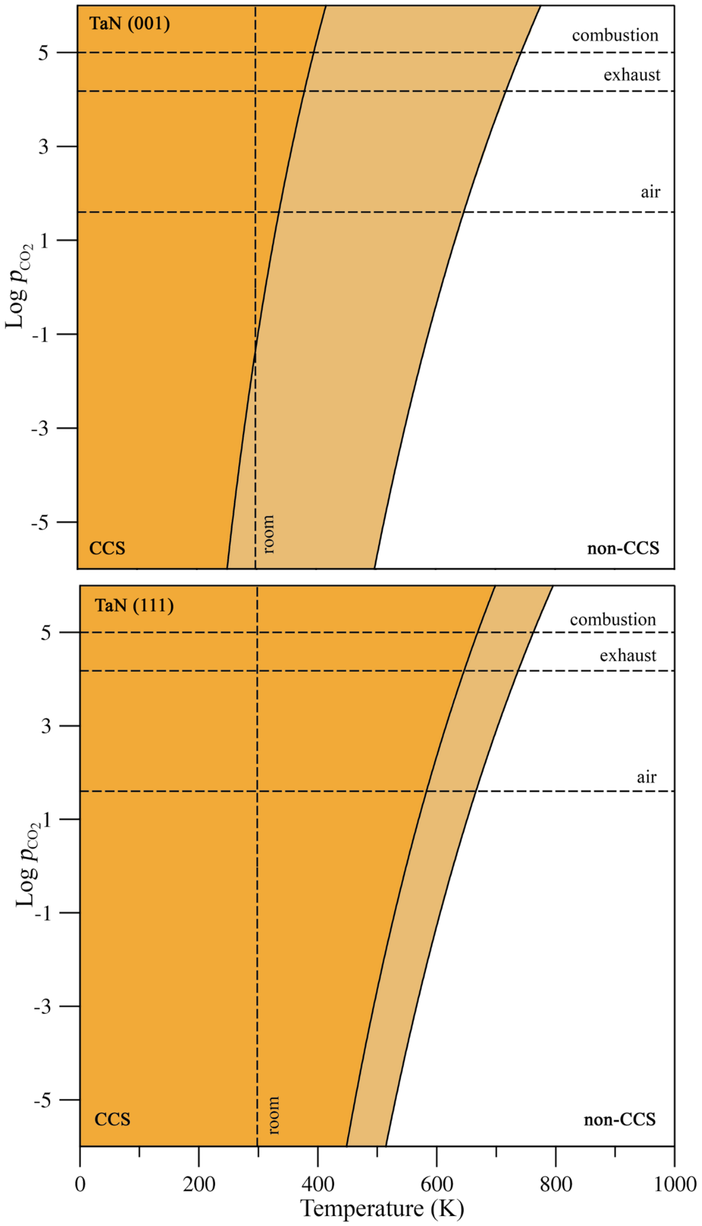

Fig S14. CCS kinetic phase diagrams of CCS) kinetic phase diagrams of Cr-derived MXenes with stoichiometries Cr_2N, Cr_3N_2, and Cr_4N_3.

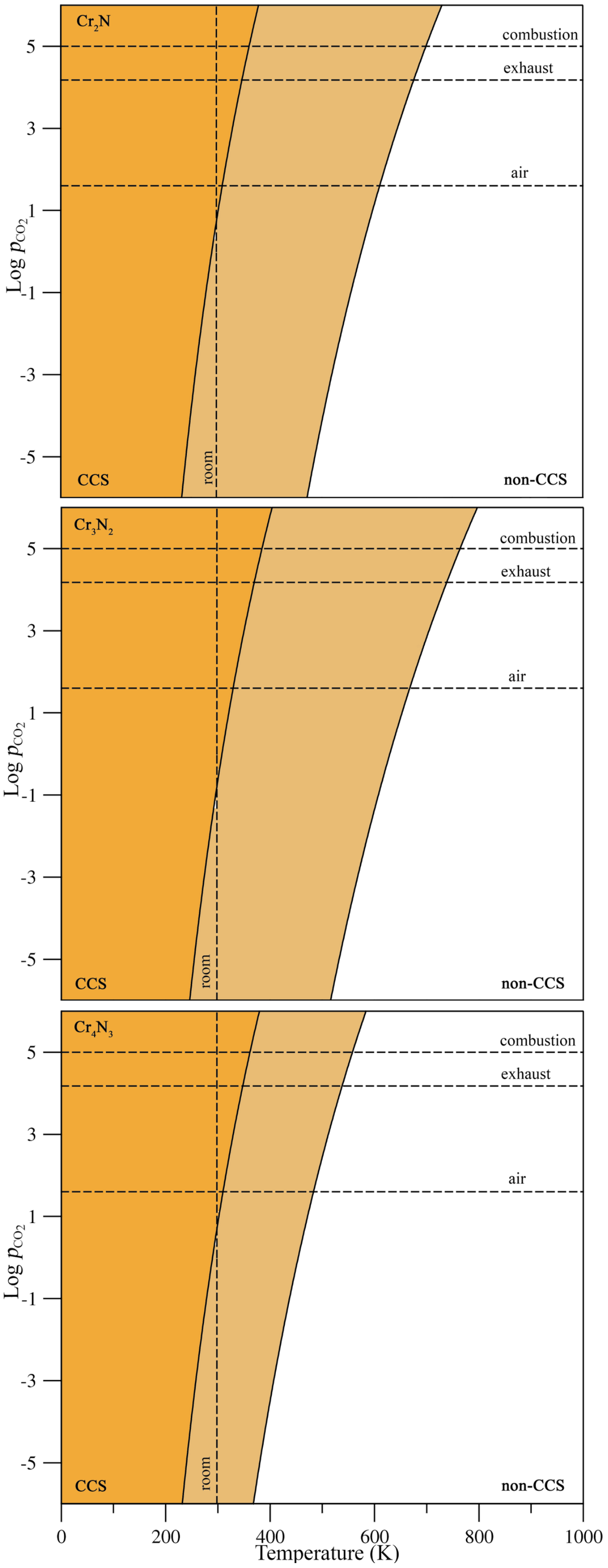

Fig S15. CCS kinetic phase diagrams of CrN (001) and (111) surfaces.

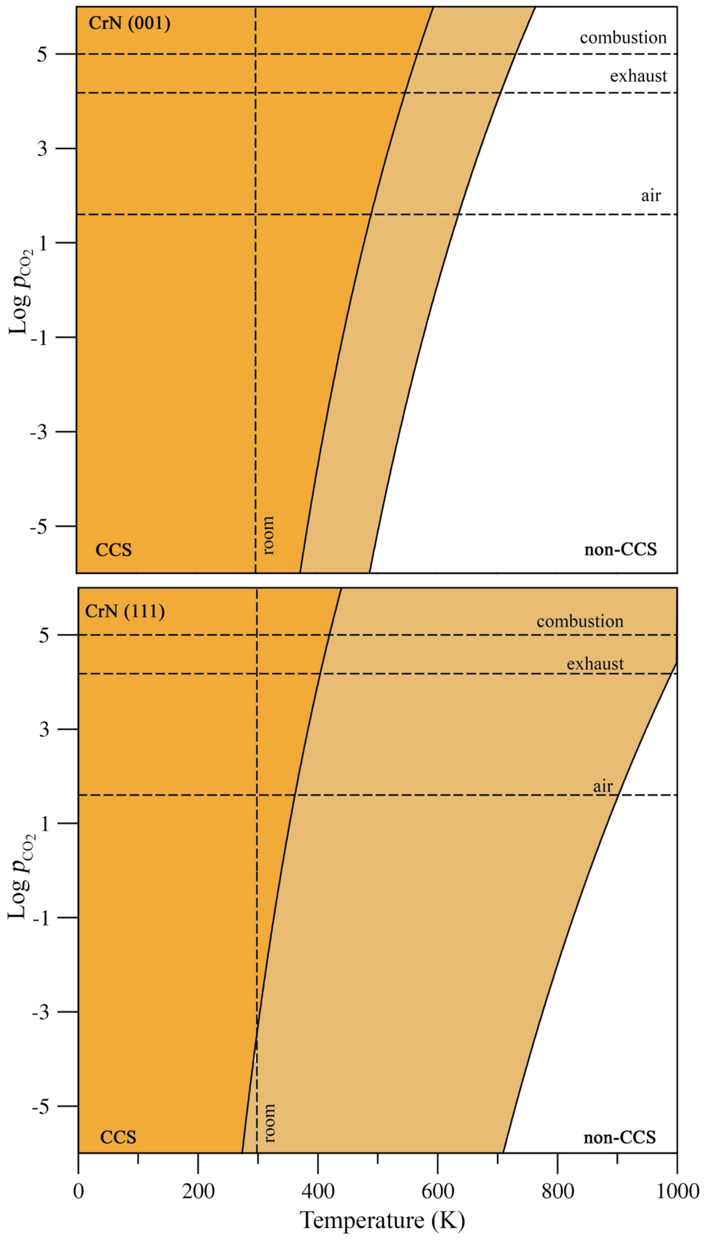

Fig S16. CCS kinetic phase diagrams of CCS) kinetic phase diagrams of Mo-derived MXenes with stoichiometries Mo_2N, Mo_3N_2, and Mo_4N_3.

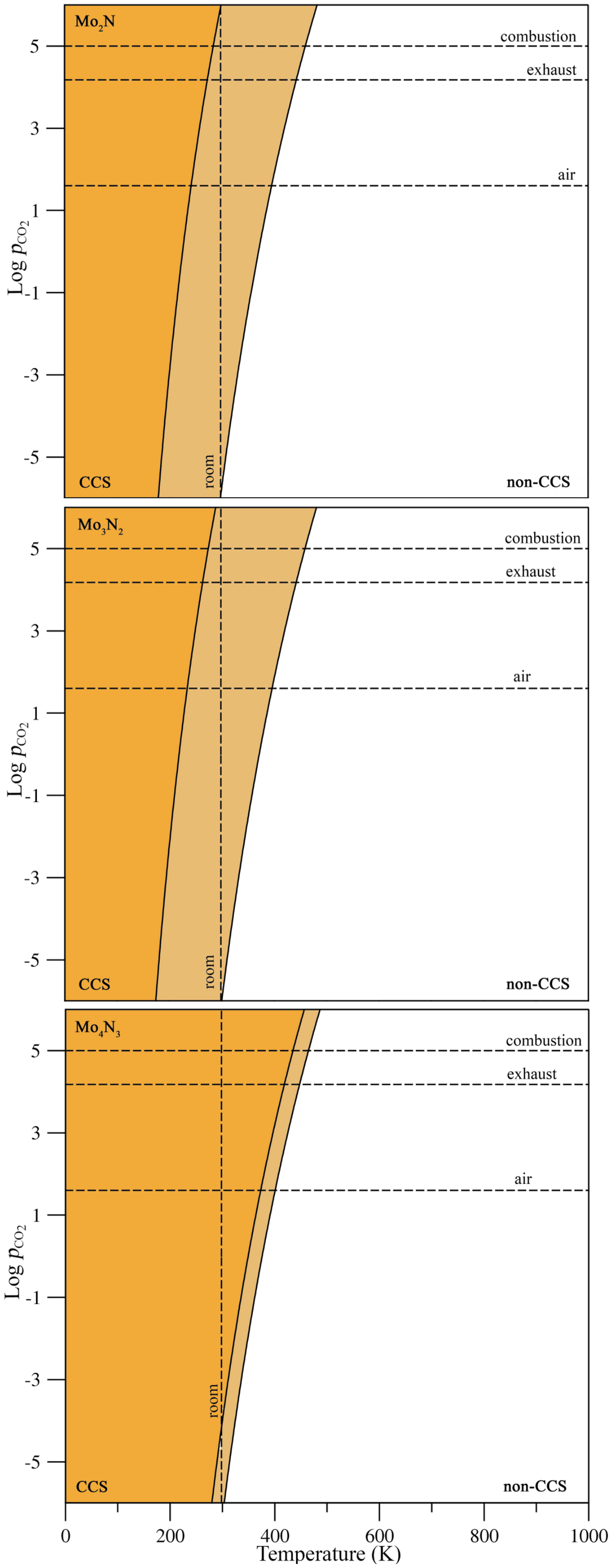

Fig S17. CCS kinetic phase diagrams of MoN (001) and (111) surfaces.

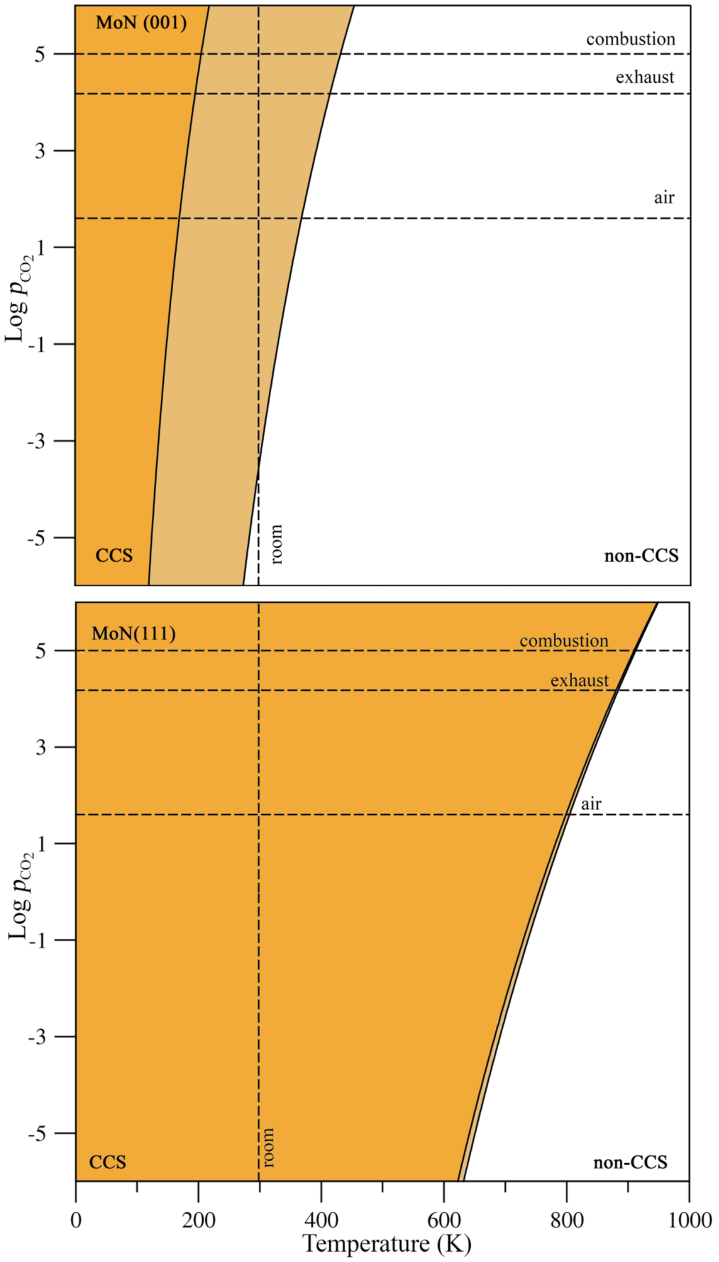

Fig S18. CCS kinetic phase diagrams of CCS) kinetic phase diagrams of W-derived MXenes with stoichiometries W_2N, W_3N_2, and W_4N_3.

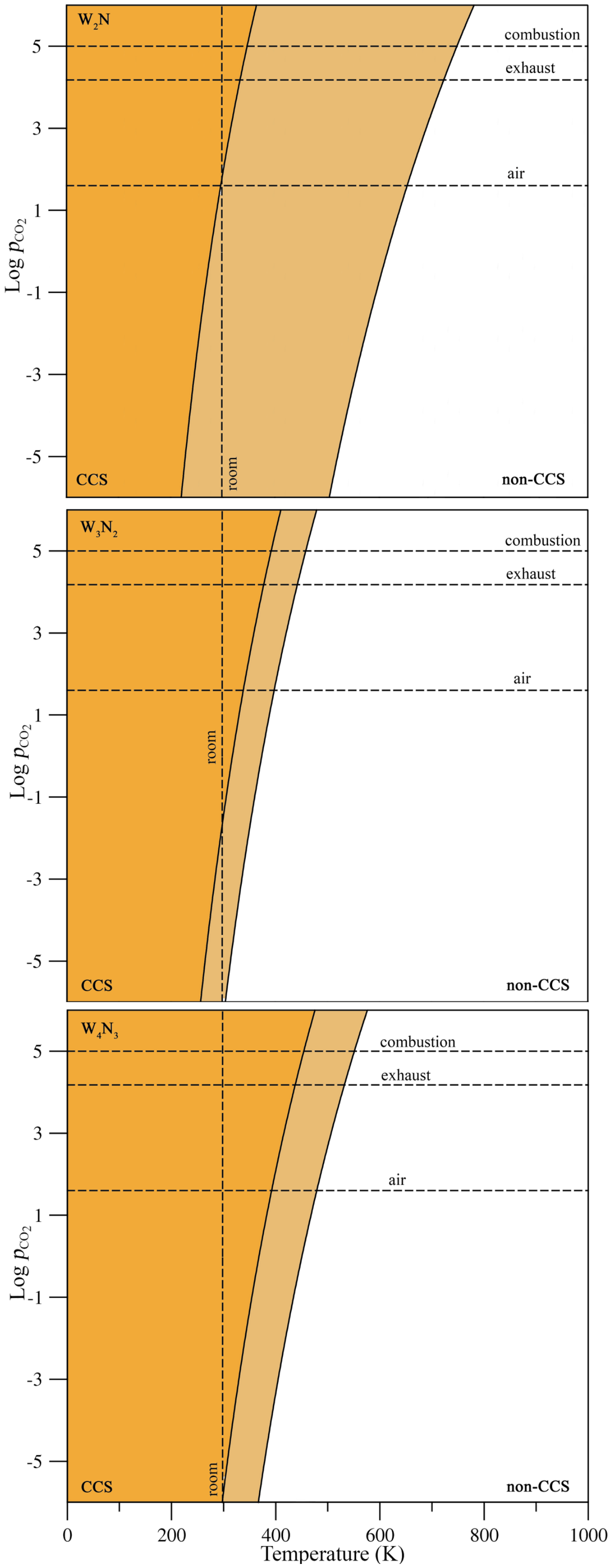

Fig S19. CCS kinetic phase diagrams of WN (001) and (111) surfaces.

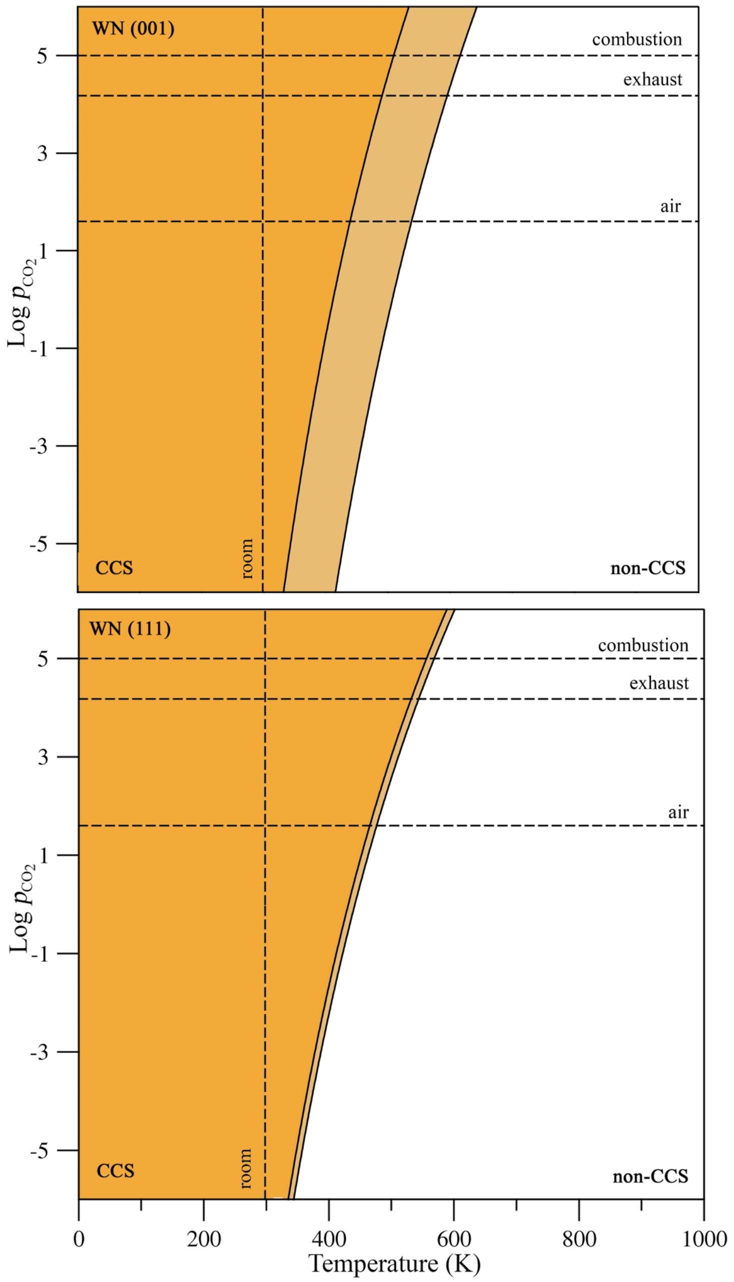

Supplement: Supplementary file 1 — Supporting Information [file CPHC-22-2456-s001.pdf]
